# Regioisomeric thieno[3,4-*d*]thiazole-based A-Q-D-Q-A-type NIR acceptors for efficient non-fullerene organic solar cells†

**DOI:** 10.1039/d4ra01513d

**Published:** 2024-04-04

**Authors:** Tahseen Iqbal, Shaoming Sun, Kerui Liu, Xiaozhang Zhu

**Affiliations:** a Beijing National Laboratory for Molecular Sciences, CAS Key Laboratory of Organic Solids, Institute of Chemistry Chinese Academy of Sciences Beijing 100190 China xzzhu@iccas.ac.cn; b University of Chinese Academy of Sciences Beijing 100049 China

## Abstract

This study explores the potential of regioisomeric quinoidal-resonance π-spacers in designing near-infrared (NIR) non-fullerene acceptors (NFAs) for high-performance organic solar cell devices. Adopting thienothiazole as the π-spacer, two new isomeric A-Q-D-Q-A NFAs, TzN-S and TzS-S, are designed and synthesized. Both NFAs demonstrate a broad spectral response extended to the NIR region. However, they exhibit different photovoltaic properties when they were mixed with the PCE10 donor to fabricate respective solar cells. The optimal device of TzS-S achieves a PCE of 10.75%, much higher than that of TzN-S based ones (6.13%). The more favorable energetic offset and better molecular packing contribute to the better charge generation and transport, which explains the relative superiority of TzS-S NFA. This work sheds new light on the regioisomeric effect of component materials for optoelectronic applications.

## Abstract 2

With better phase separation in film morphology, regioisomeric non-fullerene acceptor TzS-S shows 1.5 times higher PCE and 20 times greater electron mobilities than TzN-S, indicating the role of isomeric conformations in photovoltaic performance.

## Graphical abstract

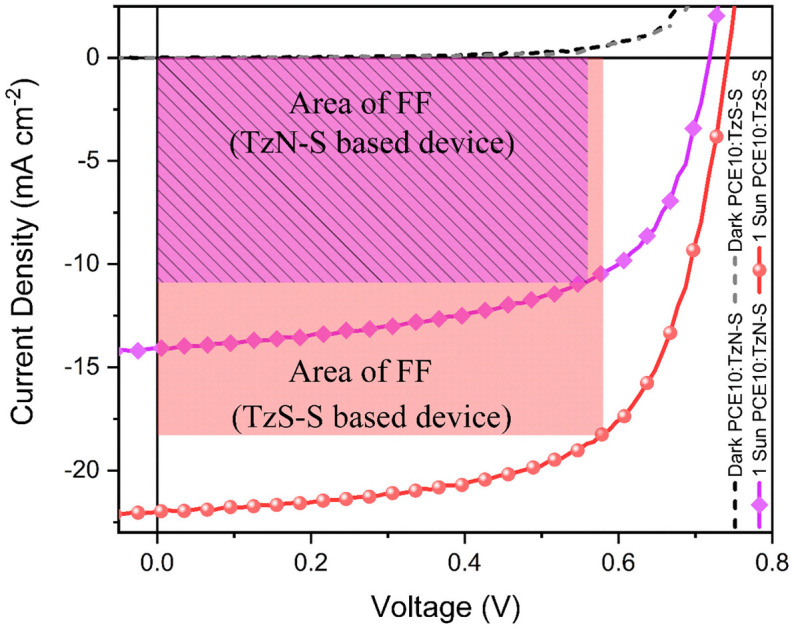

## Introduction

Organic solar cells (OSCs) exhibit higher potential to reduce CO_2_e (carbon dioxide emissions) compared to other solar technologies.^1^ Moreover, OSCs attract considerable research attention for their cost-effective fabrication,^2^ large area printing, flexibility, light weight, and semitransparency.^3^ Typical bulk-heterojunction (BHJ) OSCs comprise two mixed components of different energy levels and bandgaps, *i*.*e*., a p-type donor and n-type acceptor. The energetic difference between donors and acceptors induces a potential which results in charge dissociation of excitons at the donor–acceptor (D–A) interface, generated under solar irradiation. The resulting charge carriers are subsequently collected at respective electrodes. Initially, photovoltaic studies focused on solar cells widely incorporating fullerene based acceptors which were blended with a variety of well-developed donor materials to construct efficient BHJs. Due to the absorption limitation of fullerene-based acceptors, the power conversion efficiency (PCE) of OSCs was limited, which triggered the exploration of non-fullerene acceptors (NFAs). To date, NFA-based OSCs^4–6^ have shown a dramatic increase in the efficiency development with state-of-the-art records approaching ∼20%.^7^

Benefiting from the push–pull hybridization, most NFAs display an extended absorption and can even reach the near-infrared region (NIR) in the solar spectrum. The absorption of photons within acceptors leads to the generation of excitons and subsequent dissociation into free carriers *via* Channel-II or n-type excitation, which maximizes the overall photocurrent density for OSC devices. A promising candidate for non-fullerene acceptors is the A-D-A type NFA, which comprises a central donor (D) core as an electron (e)-rich unit and two peripheral e-deficient acceptor (A) units. This architecture is advantageous because (i) shift of absorption spectra to the NIR region and tuning of frontier molecular orbitals (FMOs) by selecting suitable D and A units is easily achieved; (ii) lowest unoccupied molecular orbital (LUMO) and highest occupied molecular orbital (HOMO) are generally localized at A and D units, respectively. Therefore, any structural change in either unit may independently change the energy level of FMOs; (iii) A-D-A type NFAs are easy to synthesize; (iv) batch-to-batch variation of properties like polydispersity, molecular weight and purity is not observed, as opposite to the polymers; and (v) A-D-A type molecular configuration and molecular alignment parallel to the interface, induce energy level bending at D–A interface which decreases the energy difference between charge transfer (CT) and charge separation (CS) states, thus facilitating the formation of free charge carriers.^8^

Previously, researchers^9–12^ have explored the usage of fluorene and carbazole cores for designing A-D-A type NFAs. However, introducing various fluorene D units led to devices with low fill factor (FF) and poor performance. Therefore, optimal indaceno[1,2-*b*:5,6-*b*′]dithiophene (IDT)^13^ and indacenodithieno[3,2-*b*]thiophene (IDTT) cores for narrow-band gap and strongly absorbing NFAs were developed. IDT based NFAs were found to have improved photon absorption and conjugation by stronger push–pull character; along with higher aggregation and phase separation tendency to form percolating networks at favorable length scales in the blends.^9^ In addition, diketopyrrolopyrrole (DPP), rhodamine, and indandione derivatives were frequently used as electron-withdrawing units for A-D-A architecture. Lin *et al.* reported IDT based A-D-A NFA, IC-C6IDT-IC, with an optimized efficiency of 8.71%.^14^ Introducing a π-spacer between the donor and acceptor units allows the better tuning of FMOs and band gap of designed materials. In 2014, Zhan *et al.* introduced electron-donating thiophene spacers and designed an A-π-D-π-A NFA IEIC, with resultant PCE of 6.31%.^15^

**Fig. 1:**
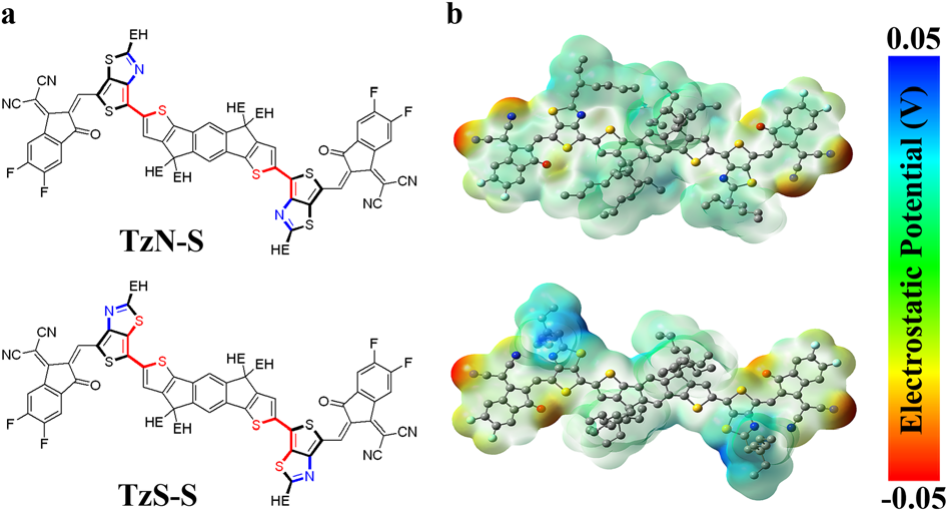
(a) Molecular structures and (b) electrostatic potential mapping for designed TzN-S and TzS-S. Higher charge densities are associated with more negative electrostatic potential regions (red), whereas negative charge densities (hole character) are associated with positive electrostatic potential regions (blue). Gaussian was used to perform the simulations with visualization obtained from GaussView.

Here in this manuscript, we designed and synthesized two new IDT based A-Q-D-Q-A NFAs, with 2-(5,6-Difluoro-3-oxo-2,3-dihydro-1*H*-inden-1-ylidene)malononitrile (2FIC) as the acceptor (A) unit, and thienothiazole (TTz, Q) as the unique π-spacer to stabilize the quinoidal character of molecules. To gain favorable morphology, the IDT core was decorated with ethylhexyl (EH) groups, which also help improve the solubility. The design produces two isomeric NFAs, named as TzN-S and TzS-S, as shown in Fig. 1a. Detailed studies on the synthesis of these isomers, along with thorough performance evaluation for photovoltaic application, have been investigated.

## Results and discussion

### Material synthesis and characterization

**Scheme 1:**
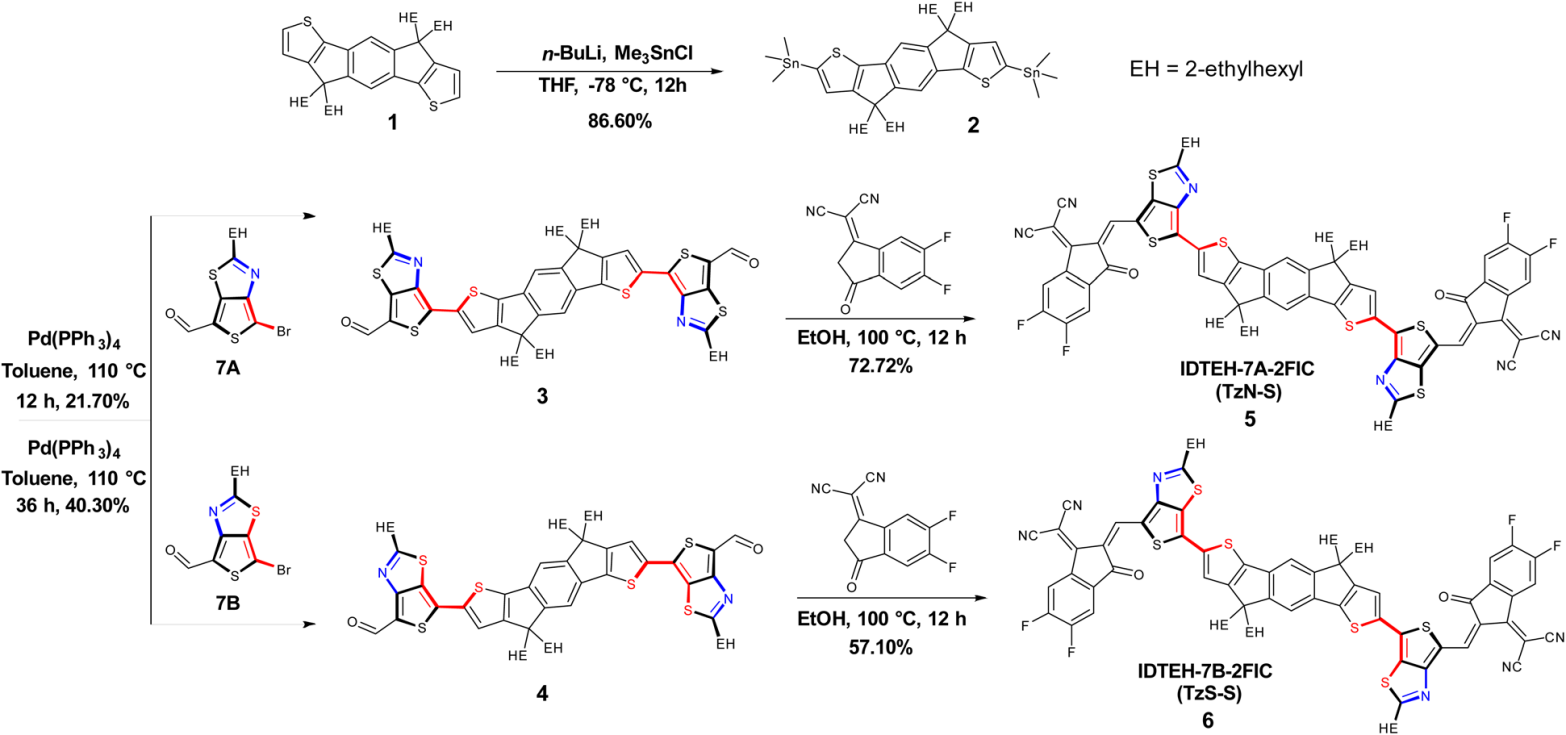
Synthesis of isomeric TzN-S and TzS-S.

The synthetic studies were carried out starting from the stannyltion^16^ of commercially available 4,4,9,9-tetrakis(2-ethylhexyl)-4,9-dihydro-*s*-indaceno[1,2-*b*:5,6-*b*′]dithiophene (1). The resulting bis-stannylated product 2 was cross-coupled^17^ with 7A or 7B to obtain respective precursors, IDTEH-7A (3) and IDTEH-7B (4). 7A and 7B were prepared by the formylation of 4,6-dibromo-2-(2-ethylhexyl)thieno[3,4-*d*]thiazole.^18^ Finally, the target NFAs, 5 and 6, were obtained by Knoevenagel condensation of respective precursors with the 2FIC acceptor units. The synthetic routes of the two NFAs are shown in Scheme 1. The incorporation of TTz as a π-bridge produces two isomeric NFAs: TzN-S with the nitrogen atom of thiazole facing the sulfur atom of thiophene on IDT backbone; and TzS-S, with the S of thiazole facing the S on IDT backbone. Both isomeric NFAs readily dissolve in common organic solvents such as chloroform (CF), chlorobenzene (CB) and 1,2-dichlorobenzene (DCB), which favors the solution processing in device preparation. Complete structural characterization of the newly synthesized materials, were carried out and shown in the ESI (ESI).†

### Optical and electrochemical properties

**Fig. 2:**
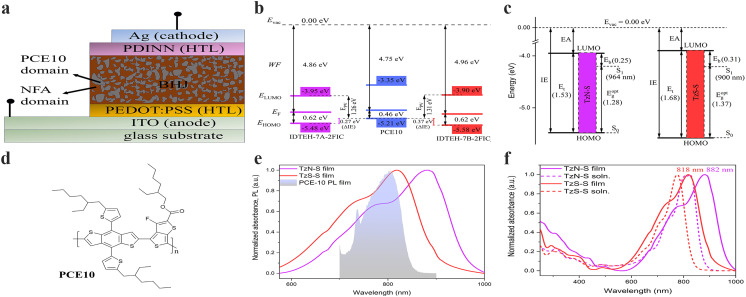
A simplified schematic representing (a) schematic illustration of a conventional OSC device; (b) energetic offsets (ΔIE) and *E*_pv_ in the D–A heterojunction of OSC devices; (c) the transport gap (*E*_t_), optical gap ( *E*^opt^_g_) and estimated binding energy (*E*_b_) of TzN-S and TzS-S); (d) the molecular structure of PCE10 donor material used in this study; (e) spectral overlap between photoluminescence (PL) of donor and absorption of regiomeric acceptors in thin film state; and (f) normalized UV-vis-NIR spectra in both solution and solid state. The given absorption values correspond to *λ*_max_ in thin film state. Furthermore, IE/EA from UPS/LE-IPES spectra were converted to valence and conduction band energies as reported earlier:^35^ IE = −*E*_HOMO_; EA = −*E*_LUMO_.

The optical behaviour of TzN-S and TzS-S in solution and film states was investigated by UV-vis-NIR absorption and emission spectroscopy (Fig. 2 and Table 1). Both NFAs exhibited extended absorption to near 1000 nm. The less intense, lower wavelength (200–500 nm) absorption bands may arise from the localized electronic π–π* transitions in the conjugated backbone/aromatic rings. However, more intense and higher wavelength (500–1000 nm) 0–0 vibronic absorption bands may be related to the π–π* electronic transitions associated with intramolecular charge transfer (ICT) between IDT and 2FIC.^19,20^ The ICT effect is stronger in TzN-S as its absorption edge is red-shifted by 39 nm, larger than that of TzS-S in solution state.^21^ Compared to the absorption in solution, both TzN-S and TzS-S films display obvious bathochromic shifts in their ICT absorption bands, which may be ascribed to the enhanced molecular ordering and increased intermolecular π–π interactions in solid states.^22^ The comparatively red-shifted ICT band of TzN-S thin film than that of TzS-S film indicates a higher degree of planarity, which is induced by the respective isomeric π-spacer. Aggregation takes place in both solution and film states of TzN-S and TzS-S, as indicated by the presence of shoulder peaks (0–1 transitions). Kasha theory differentiates the J-aggregates from H-aggregates by comparing solution and thin film state absorption spectra; *i.e.*, J-aggregates (H-aggregates) give rise to aggregation peaks red-shifted (blue-shifted) in thin films than in solution. However, the theory considers the Coulomb intermolecular interactions (through space) only, and doesn't account for the fine aromatic-quinoidal vibronic coupling which also contributes to the photophysical properties of soft materials. Spano introduced another way to differentiate aggregation types by comparing the intensity ratio of first two absorption peaks (*I*^0-0^/*I*^0-1^) relative to the unity.^20^ With *I*^0-0^/*I*^0-1^ > 1, both TzN-S (1.28) and TzS-S (1.37) exhibit J-aggregation in film states. This pre-aggregation may be ascribed to the strong non-covalent/van der Waals interactions^23^ like F⋯S, C–H⋯F, C–F⋯π, N⋯F, *etc*. Moreover, the *λ*^film^_onset_ of TzN-S occurs at ∼964 nm, redshifted by 64 nm to that of TzS-S (900 nm), which also suggests stronger aggregation in TzN-S than TzS-S film. Both TzN-S and TzS-S films complements well with PCE10 on spectral absorption which enables superior light-harvesting property, making these isomers good acceptor candidates for OSCs. The maximum absorption coefficient of TzS-S in film state is 4.80 × 10^5^ cm^−1^, higher than that of TzN-S (3.36 × 10^5^ cm^−1^), which may benefit the acquisition of better *J*_sc_ and PCE of OSC devices.

**Table 1:** Photophysical data and PES-derived electronic energy values of the studied materials

Materials	Optical properties							Electronic properties			
	*λ* ^abs,soln^_max_ a [nm]	10^5^ × *ε*^soln^_max_ [M^−1^ cm^−1^]	*λ* ^abs,film^_max_ b [nm]	Aggregation	*λ* ^film^_onset_ [nm]	*E* ^opt,film^_g_ c [eV]	*δν* d [nm]	*E*_LUMO_ [eV]^e^	*E*_HOMO_ [eV]^f^	*E*_t_ g [eV]	*E*_b_ h [eV]
TzN-S	823	3.60	882	J-type	964	1.28	83	−3.95	−5.48	1.53	0.25
TzS-S	776	4.62	818	J-type	900	1.37	69	−3.90	−5.58	1.68	0.31
PCE10	691	0.27	702	J-type	776	1.60	104	−3.35	−5.21	1.86	0.26

a 10^−5^ M in CHCl_3_.

b Thin film as spin-coated from CHCl_3_ solution (10 mg mL^−1^) at 3000 rpm for 0.5 min.

c Anticipated from onset of absorption band in charge transfer region of absorption spectrum, using: *E*^opt,film^_g_ = 1240/*λ*^film^_onset_.

d Stokes shift.

e EA estimated from LE-IPES and correlated as EA = −*E*_LUMO_ (Koopmans' theorem).^33^

f IE estimated from UPS and correlated as IE = −*E*_HOMO_ (Koops theorem).

g *E*_t_ = *E*_LUMO_ − *E*_HOMO_, transport gap.

h *E*_b_ = *E*_t_ − *E*^opt,film^_g_, binding energy.

Emission spectra of NFAs were recorded and compared with the corresponding absorption spectra to estimate Stokes shift (Fig. S1†), which is associated with the non-radiative energy losses.^24^ Compared to the TzN-S film (83 nm), the TzS-S film exhibits a smaller Stokes shift (69 nm), which indicates lower non-radiative energy losses in its solid state. Therefore, TzS-S is expected to show better energy conversion than TzN-S.

The electrochemical response of materials understudy was investigated by their first oxidation and reduction waves obtained from cyclic voltammetry (CV). The cyclic voltammograms of both regiomers (Fig. S2†) display reversible oxidation waves in contrast to the irreversibility found in their reduction waves. We tentatively assign the irreversible waves to the reduction process in 2FIC unit. This irreversibility arises when the anionic radical specie, resulted from electron transfer to the 2FIC unit, may be either electropolymerized or decomposed on the surface of electrode.^25,26^ Lower reduction potential of TzN-S (*E*_Pc_ = −0.51 V *vs.* Ag/AgCl) might result from its better electron-accepting characteristics due to the lower LUMO energy level,^27^ compared to that of TzS-S. On the other hand, the reversible oxidation waves could be ascribed to the oxidation process in the main chain containing e-rich donor core, IDTEH.^28^ Oxidation process generates a radical cation [IDTEH-7A/7B]˙^+^ in the main chain, which is stabilized by the thienyl/thiophene functionalities.^29^ The stability of this radical cation appears as a return peak (cathodic peak) in the voltammogram, thereby forming a closed current–voltage loop (reversible oxidation wave). Compared to TzN-S, more positive oxidation potential (*E*^1/2^_ox_) of TzS-S (1.24 V *vs.* Ag/AgCl) indicates decreased electron density in its donor core which makes it less susceptible to oxidation^27,30^ and more stable, electrochemically. The onset of first oxidation and first reduction wave of each isomer was used to calculate the energy levels of HOMO and LUMO, respectively. Exciton dissociation requires sufficiently large energetic offset at the D–A interface in a BHJ soar cell.^31^ The LUMO offset (Δ*E*_LUMO_ = *E*_LUMO(A)_ − *E*_LUMO(D)_) regulates the electron transfer while the HOMO offset (Δ*E*_HOMO_ = *E*_HOMO(D)_ − *E*_HOMO(A)_) governs the hole transfer across the D–A interface. A recent study has proposed a minimum Δ*E*_HOMO_ of 0.5 eV necessary for complete dissociation of excitons into free carriers in low-bandgap NFA OSCs. Ultraviolet photoelectron spectroscopy (UPS) and low-energy inverse photoelectron spectroscopy (LE-IPES) provide reliable experimental approximations for IE and EA, respectively.^32,33^ Therefore, we used UPS and LE-IPES to estimate IE offset (ΔIE) and EA offset (ΔEA), respectively. In this study (Fig. 2a and S3†), PCE10:TzS-S exhibited a ΔIE of 0.37 eV, higher than that of PCE:TzN-S (ΔIE = 0.27 eV), which may counterbalance the high-lying CT state energy due to interfacial energy level bending and thereby improve free charge conversion^34^ and PCE. Moreover, we applied the B3LYP/6-31G(d.p) method to perform DFT analysis and used the resultant HOMO/LUMO energies (Fig. S8†) for further correlations.

### Photovoltaic performance

**Fig. 3:**
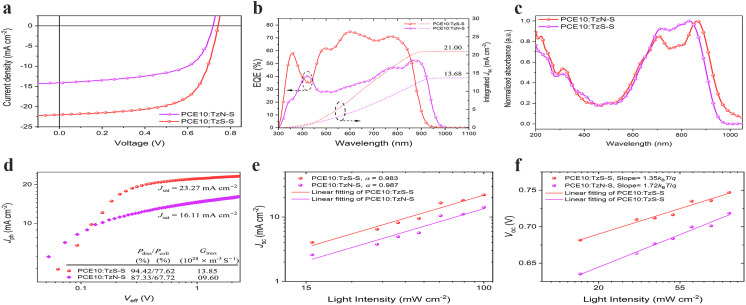
(a) *J*–*V* curves and (b) corresponding EQE spectra of the optimal PCE10: acceptor based OSC devices. (c) Normalized UV-vis-NIR absorption spectra of the blend films understudy. (d) *J*_Ph_–*V*_eff_ characteristics, (e) light intensity dependence of *J*_sc_ showing negligible bimolecular recombination, and (f) variation of *V*_oc_ with light intensity for PCE10:acceptor based fabricated devices.

The photovoltaic properties of regio-isomeric TzN-S and TzS-S were investigated by making BHJ devices with a conventional architecture: ITO/PEDOT:PSS/PCE10:acceptor/PDINN/Ag. Both photovoltaic blends were processed in chloroform. The optimized weight ratio of PCE10 : acceptors is 1 : 1.5. Fig. 3a displays the current density–voltage (*J*–*V*) curves of OSC devices under optimized condition with detailed device parameters listed in Table 2. Notably, the photovoltaic performance of TzS-S surpasses that of TzN-S isomer. The TzN-S based device exhibits a low PCE of 6.13% with a *V*_oc_ of 0.70 V, a *J*_sc_ of 14.27 mA cm^−2^ and FF of 61.36%; however, TzS-S exhibits a significantly improved PCE of 10.75% with a *J*_sc_ of 22.09 mA cm^−2^, a *V*_oc_ of 0.75 V and FF of 65.14%. The smaller energetic offset (ΔIE = 0.27 eV) leads to a decreased CT state dissociation efficiency and reduced photocurrent generation,^36^ which may hamper the efficiency of PCE10:TzN-S blend. The EQE curves are shown in Fig. 3b. The TzS-S based device displays a higher EQE of over 70% from 555 to 800 nm with maximum approaching 74.86%. The integrated *J*_sc_ value, obtained from respective EQE curve, is higher for TzS-S based device (21 mA cm^−2^) compared to that of TzN-S based device (13.27 mA cm^−2^), which are within 5% error to that extracted by *J*–*V* measurement, suggesting the reliability of data.^17^

**Table 2:** Photovoltaic performance of the optimal OSCs based on PCE10: acceptors

BHJ^a^	*V*_oc_ [V]	*J*_sc_ [mA cm^−2^]	*J*_sc_,_cal_^b^ [mA cm^−2^]	FF [%]	PCE^c^ [%]	*E*_g,EQE_/*q* [V]	Δ*V*_oc_ [V]
TzN-S	0.70	14.27	13.68	61.36	6.13	1.32	0.62
	(0.70 ± 0.01)	(14.16 ± 0.45)		(60.06 ± 2.05)	(6.00 ± 0.21)		
TzS-S	0.75	22.09	21.00	65.14	10.75	1.43	0.68
	(0.74 ± 0.003)	(21.56 ± 0.35)		(65.11 ± 0.83)	(10.45 ± 0.13)		

a PCE10 was used as donor material to prepare BHJ mixture with donor : acceptor weight ratio of 1 : 1.5.

b The integral *J*_sc_ extracted from the EQE curves.

c The average values and standard deviations in parentheses, represent the statistical data obtained from eighteen independent cells.

### Device physics: charge transport, exciton dissociation and charge recombination

Photocurrent (*J*_ph_) generation in OSC devices, initiates by the optical absorption in the constituent D and/or A units, giving rise to spin-singlet spatially bound electron–hole pairs, Frenkel excitons. The resulting excitons diffuse toward D–A interface where dissociation takes place *via* ultrafast electron transfer to A and hole transfer to D component in the BHJ. These electrons and holes are not completely separated, and bound by Coulomb forces of attraction at D–A interface, forming charge-transfer (CT, D^+^–A^−^) excited states. These CT states, if not lost due to geminate recombination phenomena, are further dissociated to form CS states, where free charge carriers are generated. Transport of these carriers to the respective electrodes, generates *J*_ph_ in OSC devices.^37^

We studied the exciton dissociation and charge collection for optimal devices by plotting *J*_ph_ as a function of effective voltage (*V*_eff_). Defined as *J*_ph_ = *J*_L_ − *J*_D_, the *J*_ph_ is experimentally determined under 1 sun simulated light (*J*_L_) corrected for the dark current density (*J*_D_); and *V*_eff_ is given by *V*_eff_ = *V*_0_ − *V*_bias_, where *V*_0_ is the compensation voltage at which *J*_ph_ = 0 while *V*_bias_ is the externally applied bias voltage. At high *V*_eff_ (>2.0 V), majority photogenerated excitons dissociate into free charge carriers and are effectively collected. *J*_ph_ in both devices increases linearly in the low *V*_eff_ regime conversely to the saturation regime (*V*_eff_ > 2.0 V) where saturated current densities (*J*_sat_), *i.e.*, 2.28 V for TzN-S and 2.35 V for TzS-S based OSC device, were observed. The exciton dissociation probability (*P*_diss_ = *J*_sc_/*J*_sat_) under short circuit conditions (*V* = 0 V) and charge collection probability (*P*_coll_ = *J*_max_/*J*_sat_) at maximum power points were also determined. Under short circuit conditions, ∼94% of the e–h pairs were dissociated in TzS-S based device, higher than that of TzN-S based device (∼87%). The *P*_coll_ at maximum power point decreased to 77.62% and 67.72% for TzS-S and to for TzN-S based device respectively. We also estimated the maximum generation rate (*G*_max_) for free charge carrier in both devices according to *J*_sat_ = *qG*_max_*L*, where *q* is the elementary charge and *L* is the active layer thickness. The TzS-S-based device shows a *G*_max_ of 13.85 × 10^28^ m^−3^ S^−1^, higher than that of TzN-S based device (9.60 × 10^28^ m^−3^ S^−1^). The results show that compared to TzN-S based device, the TzS-S based device exhibits more efficient exciton dissociation and more effective charge collection. Therefore, due to better light absorption and higher production of excitons^38^ in its active layer, the higher *J*_sc_ and FF of the TzS-S based device is understandable.

We investigated the charge recombination of two devices by correlating the varying light intensity (*P*_light_) with *V*_oc_ and/or *J*_sc_. According to *V*_oc_ ∝ *S* ln(*P*_light_), the slope (*S* = *n* × *KT*/*q*) differentiates the recombination processes as *n* = 0.5 for surface recombination, *n* = 1.0 for bimolecular recombination, and *n* = 2.0 for monomolecular or trap state-induced recombination (*K* represents the Boltzmann constant, *T* is the thermodynamic temperature, *q* is the elementary charge, and *n* denotes the ideality factor).^39^ The slope value of 1.35*KT*/*q*, obtained for TzS-S based device, correlates the loss of charge carriers in its photoactive layer to the bimolecular recombination (*R*_bm_), in contrast to the dominant trap state-induced (*R*_t,s_) recombination phenomena which cause efficiency loss in TzN-S based device (1.72*KT*/*q*). Moreover, the degree of *R*_bm_ can be anticipated using the power law *J*_sc_ ∝ (*P*_light_)^*α*^ where there are no *R*_bm_ losses if *α* = 1, indicating collection of all charge carriers at respective electrodes.^40^ Contrarily, the extent of *R*_bm_ is related to the *α* < 1; the further lower it is from unity, the higher is the *R*_bm_. TzS-S based device was found to have *α* = 0.983, which is indicative of negligible *R*_bm_ losses in its photoactive layer; thereby leading to higher *J*_sc_ and FF compared to TzN-S based device.

Mobility balance serves as a key parameter in achieving high efficiency OSC devices. The imbalanced carrier mobility builds-up space charge in the device due to accumulation of carriers with lower mobility, and promotes recombination; thereby leading to a significant drop in *J*_sc_ and FF.^41^ Conversely, the balanced mobility of charge carriers contributes effectively in efficiency enhancement^42^ of OSC devices. We evaluated the charge transport behavior using a space-charge limited current (SCLC) method with mobilities calculated by fitted *J*^0.5^–*V* curves of respective electron- or hole-only devices. The PCE10:TzS-S blend demonstrated relatively higher and more balanced mobility (*μ*_e_/*μ*_h_ = 9.26 × 10^−4^ cm^−2^ V^−1^ s^−1^/7.02 × 10^−4^ cm^−2^ V^−1^ s^−1^ = 1.32), which contributes to the obtained higher *J*_sc_ and FF.

### Topographical and morphological studies

Surface topography of the blend films was studied by images collected from atomic force microscope (AFM). Both surfaces display scattered aggregates in their thin films. Rougher surfaces are generally beneficial since the interface area for facile charge separation may be increased. Besides, increased internal light absorption may be resulted due to nano-scale textures.^47–49^ The surface of PCE10:TzS-S blend film shows a *R*_q_ of 5.32 nm, smaller than that of PCE10:TzN-S (10.10 nm). The *R*_q_ for TzN-S or TzS-S blends associates with the relative size of their aggregates, which were observed further by transmission electron microscopy (TEM). As shown in Fig. S6a,† the TzN-S blend film features a network with non-uniform large domains, reflecting poor miscibility within the blend which may lead to large phase separation and reduced interfacial areas for charge separation.^49^ Kinetic Monte Carlo modeling proposed, an ideal blend morphology of active layers shall comprise domains around 10–20 nm with interpenetrating networks formed to maximize the interfacial areas.^50^ However, larger domains may increase the probability of exciton trapping and results in photocurrent loss.^51^ This can be the reason to the low performance of TzN-S blend. In contrast, nano-scaled phase separation formed in PCE10:TzS-S blend with obvious bicontinuous interpenetrating networks (Fig. S6b†). Fibrous networks are favourable as they may induce efficient exciton dissociation and facilitate charge transport by developing a suitable pathway.^52^ The small TzS-S fibrous domains increase the exciton diffusion interface with also contribute to better charge generation, which leads to higher *J*_sc_ and FF.

**Fig. 4:**
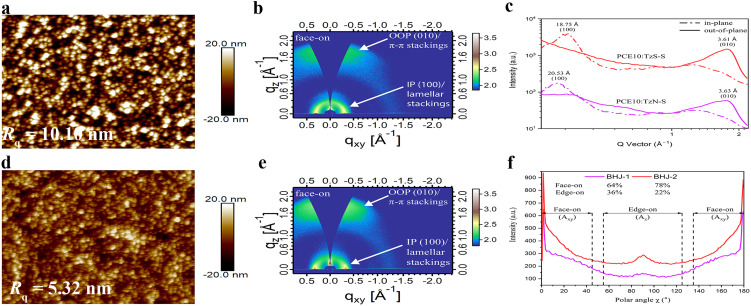
AFM images and 2D GIWAX patterns of PCE10:TzN-S (a and b) and PCE10:TzS-S (d and e) blend films along with the corresponding 1D line-cut profiles (c). Pole figure plot (f) extracted from the IP (100) lamellar scattering, each of BHJ-1 (PCE10:TzN-S) and BHJ-2 (PCE10:TzS-S) film, where polar angle (*χ*) range^43^ is defined corresponding to the face-on (*A*_*xy*_) and edge-on (*A*_*z*_) crystallites.

More detailed morphological studies were conducted by grazing-incidence wide-angle X-ray scattering (GIWAXS). The out-of-plane (OOP) Bragg reflections at |*q*| = 1.73 Å^−1^ and |*q*| = 1.74 Å^−1^ for PCE10:TzN-S and PCE10:TzS-S blends, correspond to a *d*-spacing of 3.63 Å and 3.61 Å, respectively, suggesting similar (010) π–π stacking in the OOP direction. Gaussian multipeak fit of these peaks indicates a larger crystal coherence length (CCL) within PCE10:TzN-S (Table 3). CCL reflects the crystal size or the distance over which a material preserves its order of packing. High CCL in the π–π stacking direction is desirable for effective charge transport and performance enhancement of OSC devices. CCL/d calculation helps to estimate the periodicity of a material's packing over a certain distance and its value is correlated to the average number of lamellas in the *L* length.^53^TzS-S (CCL/d = 6.86) has higher periodicity than TzN-S (CCL/d = 4.72) in the π–π direction, resulting in higher charge carrier mobilities in the blend film. The low carrier mobilities within PCE10:TzN-S is attributed to the large crystalline grain (CCL = 1.454 nm) since exciton dissociation interface decreases with the domain size increased.^45^TzS-S is more ordered compared to TzN-S, as is evident by its lower FWHM (0.23 Å^−1^) and higher scattering intensity of (010) peak (Fig. 4), which is attributed to the increased paracrystalline or increased crystallite size (CCL = 2.428 nm) resulted from dissipated amorphous regions nearby.^54^ Mixing TzS-S with PCE10 results in a FWHM value of 0.51 Å^−1^, broader than either value of pristine acceptor or donor, which reflects good intermixing. However, the lamellar space of neat polymer (*d* = 4.00 Å) decreases in PCE10: TzS-S blend (*d* = 3.62 Å), indicating the close packing^55^ of PCE10, which shows a CCL of 11.20 Å, smaller than that in PCE10:TzN-S. This small size in PCE10: TzS-S blend film may be attributed to the favourable intermolecular interactions between the π-stackings of its donor and acceptor materials, which might help in achieving a higher interface-to-volume ratio for effective charge separation and higher PCE.^56^

**Table 3:** Stacking characteristics of BHJ-1 (PCE10:TzN-S), BHJ-2 (PCE10: TzS-S), TzN-S, TzS-S, and PCE10 spin-cast thin films on PEDOT:PSS coated Si wafer

Film	IP (100) lamellar stacking					OOP (010) π–π stacking					Face-on^d^ [%]
	*q*_*xy*_	*d* a [Å]	Δ*q*^b^ [Å^−1^]	CCL^c^ [Å]	CCL/d	*q*_*z*_ [Å^−1^]	*d* a [Å]	Δ*q*^b^ [Å^−1^]	CCL^c^ [Å]	CCL/d	
BHJ-1	0.31	20.54	0.07	83.17	—	1.73	3.63	0.39	14.54	—	64.00
BHJ-2	0.34	18.76	0.08	74.42	—	1.74	3.62	0.51	11.20	—	78.00
TzN-S	0.32	19.46	0.07	84.42	4.34	1.78	3.54	0.34	16.68	4.72	—
TzS-S	0.34	18.54	0.07	81.97	4.42	1.78	3.54	0.23	24.28	6.86	—
PCE10	0.26	24.55	0.15	38.48	1.57	1.57	4.00	0.46	12.30	3.07	—

a *d*-spacings were calculated using *d* = 2π/|*q*| where |*q*| represents the reciprocal of respective peak positions (*q*_*xy*_ or *q*_*z*_);^44^ Gaussian multipeak fit was performed for IP (100) and OOP (010) stackings to find.

b Δ*q* which is the full width at half maximum (FWHM).

c CCL represents the crystal correlation length of each stacking which was estimated using Scherrer equation^45^ (CCL = 2π*K*/Δ*q*) where *K* = 2(ln 2/π)^1/2^ ≅ 0.93; however, the most often reported value of K (Scherrer constant) is ∼0.9;^46^

d Relative fraction of the face-on orientation with respect to the edge-on orientation, estimated from the pole figure analysis^43^ of (100) peak in the respective films.

Alkyl (lamellar) stackings in both blends, diffracted incident X-ray beams along IP [100] direction, as shown in 2D GIWAXS pattern. The resultant (100) peaks for both blend films broaden, showing higher intensity than those in corresponding pristine materials. Furthermore, interplanar distance of the donor decreases in both blend films, suggesting a suitable intercalation^57^ of donor alkyl chains with the alkyl side chains of TzN-S and TzS-S along IP [100] direction. However, this decrease is higher for PCE10:TzS-S film, which shows a CCL of 74.42 Å suggesting compactly lamellar packing.

The orientation of a molecular packing can be correlated to the location of the diffraction peak relative to the substrate.^44,58,59^ The (010) π–π stacking are found to be normal to the Si substrate at *q*_*z*_ = 1.57–1.78 Å^−1^ while the (100) lamellar stackings arrange themselves in the plane of Si substrate at *q*_*xy*_ = 0.26–0.31 Å^−1^. Both blend films exhibit preferential ‘face-on’ orientation. These projections pose no difference between the relative population of each orientation in the blend films. Therefore, we approximated the volume fraction of each orientation (face-on and edge-on) using the pole figure with intensity of the peak sb as a function of Azimuth/polar angle (*χ*).^60–62^ Pole figure was constructed by extracting the data from IP (100) lamellar diffraction peak. Fractions of face-on oriented crystallites were estimated from the area (*A*_*xy*_) of this peak integrated with the polar angle *χ* from 0 to 45° and 135 to 180°. Similarly, the area (*A*_*z*_) integrated with the polar angle *χ* from 55 to 125°, was related to the edge-on oriented crystallites.^43^ Analysis of this pole figure reveals that the ordered face-on crystallites in PCE10:TzS-S film (78%) are higher than in PCE10:TzN-S film (64%). The higher proportion of face-on orientation with enhanced intermolecular interactions along OOP [010] direction contributes to the higher charge carrier mobility and PCE for PCE10:TzS-S blend. Insightful information from the combined results of AFM, TEM and GIWAXS studies, has been helpful in differentiating the photovoltaic properties of both regiomers.

## Conclusions

Two NIR regioisomers are designed and synthesized based on an A-Q-D-Q-A architecture with quinoidal thienothiazole introduced as a π-bridge. The two isomers show different optical, electrochemical and photovoltaic characters. The isomer featuring a nitrogen atom on the thiazole spacer facing to the sulfur atom on the terminal thiophene, defined as TzN-S, shows an extended UV-vis absorption yet a smaller molar absorptivity in solid, compared to TzS-S, with a sulphur atom on thiazole facing to the sulfur atom on the terminal thiophene. Their photovoltaic behavior differ significantly. Compared to TzN-S (6.13%), TzS-S obtains a superior PCE of up to 10.75% with a high *J*_sc_ of 22.09 mA cm^−2^ and an FF of 65.14%. The relative performance enhancement is attributed to the improved miscibility, more favorable crystal orientation, efficient charge generation, and better electron mobility. This work provides new insights on designing high performance NIR organic semiconductors by establishing a relationship between quinoidal isomers and photovoltaic performance.

## Author contributions

X. Zhu conceived the design of target materials and acquired funding. T. Iqbal synthesized materials and fabricated devices for photovoltaic characterization. T. Iqbal performed SCLC measurements and analyzed data obtained from GIWAXS measurements. S. Sun prepared ZnO NPs for inverted OSC devices. K. Liu performed computational studies. T. Iqbal prepared the draft and X. Zhu finalized the manuscript. All authors contributed to the data analysis.

## Conflicts of interest

There are no conflicts to declare.

## Supplementary Material

RA-014-D4RA01513D-s001
