# Supplementary material for: Regioisomeric thieno[3,4-d]thiazole-based A-Q-D-Q-A-type NIR acceptors for efficient non-fullerene organic solar cells
Source: RSC Adv. 2024 Apr 4;14(16):10969–77. doi: 10.1039/d4ra01513d (PMC10993312; doi:10.1039/d4ra01513d)
Supplement: RA-014-D4RA01513D-s001 [file RA-014-D4RA01513D-s001.pdf]

Electronic Supplementary Material (ESI) for RSC Advances.
This journal is © The Royal Society of Chemistry 2024

Supporting Information

Regioisomeric thieno[3,4-*d*]thiazole-based A-Q-D-Q-A-type NIR acceptors for efficient non-fullerene organic solar cells

*Tahseen Iqbal, Shaoming Sun, Kerui Liu, and Xiaozhang Zhu**

T. Iqbal, S. Sun, K. Lui, and X. Zhu

Beijing National Laboratory for Molecular Sciences, CAS Key Laboratory of Organic Solids, Institute of Chemistry, Chinese Academy of Sciences, Beijing 100190, China

University of Chinese Academy of Sciences, Beijing100049, China

E-mail: xzzhu@iccas.ac.cn

EXPERIMENTAL SECTION

Materials and Methods. Reagents and Solvents. All commercially available reagents, unless otherwise specified, were purchased from Sigma-Aldrich, Acros, Energy Chemical Ltd., or J&K Scientific and used without further purification. THF was dried in a distillation assembly using Na/benzophenone.

Analytics and Instruments. Unless otherwise stated, the pre-dried glassware was assembled, heated under vacuum, and purged with N_2 under standard Schlenk technique prior to running a reaction in inert atmosphere. TLC analyses were performed using silica gel-coated glass plates, with fluorescence undicator (Merck 60 F_{254}). TLC spots were visualized under UV lamp (λ_{max} = 254 and 366 nm) while UV-inactive compounds were stained either with $KMnO_4$ solution (3.0 g $KMnO_4$, 20 g K_2CO_3, 5.0 mL (5.0%) NaOH, 300 mL H_2O) or with I_2-vapours.

^1H and $^{13}C\{^1H\}$ NMR spectral measurements were recorded on Bruker Avance III [400 MHz (^1H), 101 MHz (^{13}C)] and Avance NEO [700 MHz (^1H), 176 MHz (^{13}C)]. Chemical shifts are reported in parts per million (ppm, δ = scale) relative to the tetramethylsilane (TMS, δ = 0.00 ppm). Both ^1H NMR and ^{13}C NMR spectra are referenced against TMS as an internal standard or the residual solvent signal of $CDCl_3$ (δ (^1H) = 7.26 ppm, δ (^{13}C) = 77.16 ppm). Coupling constants (*J*) are given in Hertz [1]. Multiplicities are illustrated as s (singlet), d (doublet), t (triplet), q (quartet), p (quintet), h (heptet), m (multiplet), and br (broad).

EI, and MALDI-TOF and FTICR-MS spectra were recorded on an MALDI-FT 9.4 T, Ultraflex and Solarix instruments. These instruments were calibrated against NaTFA cluster ions at start. Calculated masses are average masses, and detected m/z values are maxima of unresolved isotope envelopes. The UV-vis-NIR and fluorescence spectral signatures were obtained using Perkin Elmer Lambda Jasco V-570 and Edinburg Instruments FP-6600 and FLS980 double-monochromator spectrophotometers.

Cyclic voltammetric measurements were recorded at 100 mV s^{-1} (source: CHI620E potentiostat) under N_2 environment, using one-compartment cell with a glassy carbon working electrode, a Pt-wire auxiliary/counter electrode, and Ag/AgCl reference electrode. 0.1 M ammonium hexafluorophosphate ($TBAPF_6$) in acetonitrile was used as a supporting electrolyte. All potentials were corrected against FC/FC^+ redox couple (4.8 eV below the vacuum level).

Hole-only and electron-only devices were prepared as ITO/PEDOT:PSS/PCE10:acceptor/MoO_3/Ag and ITO/ZnO Sg/PCE10:acceptors/PDINN/Ag, respectively. The charge carrier mobility of each blend film, was measured using following expression:

$$j = \frac{9}{8}\varepsilon_0\varepsilon_r\mu\frac{V^2}{L^3}$$

In the equation, J is the current density, ε_0 is the permittivity of free space, εr is the dielectric constant of the materials, μ is the charge carrier mobility, L is the thickness of the active layer and V is the internal voltage in the device, and V = Vappl – Vbi, where Vappl is the applied voltage to the device, Vbi is the built-in voltage due to the relative work function difference of the two electrodes.

Grazing incident wide-angle X-ray scattering (GIWAXS) was performed at the National Center for Nanoscience and Technology (NCNST), China. Following sample preparation on Si/PEDOT:PSS substrates, the measurements were taken using XEUSS, WAXS/SAXS system. The sample was irradiated at a fixed incident angle (αi) on the order of 0.18° with an X-ray energy of 8 KeV (X-ray wavelength λ = 0.154 nm), and the GIWAXS patterns were recorded with a 2D image detector (PILATUS R 300K) with the sample-to-detector distances of 130 mm.

Surface morphology of the films was collected with Bruker Multi-Mode 8 atomic force microscope (AFM) at a Peak-force tapping mode with SCANASYST-AIR probe (force constant of 0.4 N m^{-1}, tip radius of 2 nm, and resonant frequency around 100 kHz).

The UPS measurement was performed on an Axis Ultra DLD (Kratos, UK) spectrometer with an unfiltered He I (21.22 eV) excitation source and a pass energy of 5 eV. The base pressures of the analysis chamber was better than 5 $\times 10^{-10}$ Torr. A bias voltage of -9 V was applied to the sample for obtaining the secondary electrons cut-off (SECO) region. The Fermi level (EF) was calibrated from a UPS spectrum using Ar^{+} sputtered clean Au substrate. The LEIPS measurement was performed on a customized ULVAC-PHI LEIPS instrument with Bremsstrahlung isochromatic mode.

Preparation of ZnO Precursor Solution

i) KOH (56.01 g/mol * 13.5 mmol * 1.7 equiv) in 65 mL methanol, was added dropwise (in ten minutes) into the flask containing zinc acetate dihydrate (219.51 g/mol * 13.5 mmol * 1.0 equiv) in MeOH (125 mL) at 65°C. Aftert 2h and 15 min, stirring was stopped and the mixture was aged at RT for 2h. MeOH was decanted and the white precipitates of **ZnO nanoparticles** (ZnO NPs) were sonicated with methanol three times to remove the reaction residues. Finally, the ZnO NPs were suspended in methanol and the concentration was found to be 21 mg/mL.[2, 3] The thus-prepared ZnO NPS (30 μL) were statically dispensed onto the cleaned ITO substrates at 2000 rpm and annealed at 130 °C for 10 min, prior to the transfer of subtrates into glove box for OSC device preparation.

ii) The **ZnO sol gel** (128 mg mL^{-1}) was prepared by dissolving zinc acetate dihydrate ($Zn(CH_3COO)_2.2H_2O$, Aldrich, 99.9%, 1 g) and ethanolamine ($NH_2CH_2CH_2OH$, Aldrich, 99.5%, 0.28 g) in 2-methoxyethanol ($CH_3OCH_2CH_2OH$, Aldrich, 99.8%, 10 mL) under vigorous stirring for 12 h in air.[4] Following filtration through an organic nylon 6 filter (0.22 µm) and soniation for 30 min., ZnO sol gel (Sg) was dynamically dispensed (3000 rpm) onto the clean ITO substrates and annealed at 200 °C for 1 h, prior to the transfer of subtrates into glove box for OSC device preparation.[5]

Synthetic studies of **7A** and **7B** are described elsewhere (in our work to be published in near future).

Synthetic Manipulations. Stannylation of IDTEH (**1**)[6]. A 100 mL rbf containing a solution of **1** (0.5 g, 0.699 mmol, 1.0 equiv.) in THF (50 mL) under inert environment, was magnetically stirred at -78 °C, followed by dropwise addition of 1.6 M n-BuLi in hexane (0.96 mL, 1.54 mmol, 2.2 equiv.). After 02 h, 2 M trimethyltin chloride in THF (0.8 mL, 1.54 mmol, 2.2 equiv) was added and the reaction mixture was allowed to attain the RT. After RT stirring for 12 h, water (40 mL) was added to quench the reaction, followed by extraction with EtOAc (3 x 100 mL). The collected organic fractions were dried with brine (100 mL) and $MgSO_4$. The light yellow oil **2** (630.2 mg, 0.61 mmol, 87%) was obtained after concentrating the filtrate on rotavap and used in the next step without further purification.

IDTEH-7A[7]**.** A 15 ml high-pressure reaction tube (15 mL) containing **9** (0.158 g, 0.1518 mmol, 1 equiv) and **7A** (0.167 g, 0.46 mmol, 3.0 equiv) and freshly distilled toluene (12 mL) was purged with N_2 for 20 min. Following the addition of $Pd(PPh_3)_4$ (0.0088 g, 0.0076 mmol, 5 mol%) as a catalyst, the pressure tube was tightly closed and stirred in the oil bath at 110 °C. The dark red reaction mixture turned orange in less than 5 min. After completion of the reaction in 12 h, toluene was removed on rotavapor, followed by extraction of the residue with DCM. The collected fractions were dried with brine and $MgSo_4$, and concentrated under rotary evaporator. Finally, the crude mixture was passed through silica gel (200-300 mesh size) by flash column chromatography, using DCM/Hex (1/1 to 7/3) solvent system, to give bluish purple powder IDTEH7A (42.5 mg, 0.033 mmol, 22%) with R_f = 0.41 (TLC (SiO_2), DCM/Hex: 7/3, blue-colored visible round spot).

Analytical data for **IDTEH-7A:** bluish purple solid; ^{1}H NMR (700 MHz, $CDCl_3$) δ 9.78 (s, 2H), 7.73 – 7.67 (m, 2H), 7.40 (s, 2H), 3.09 (d, J = 6.8 Hz, 4H), 2.08 – 1.94 (m, 12H), 1.53 – 1.44 (m, 8H), 1.43 – 1.34 (m, 8H), 1.09 – 0.88 (m, 46H), 0.73 – 0.68 (m, 12H), 0.65 – 0.58 (m, 6H), 0.57 – 0.50 (m, 6H). ^{13}C{^{1}H} NMR (176 MHz, $CDCl_3$) δ 179.16, (156.20, 156.13, 156.12, 156.04), (154.63, 154.61, 154.58, 154.56, 154.54), (153.82, 153.79, 153.78, 153.76), 146.11, (136.11, 136.09), (135.25, 135.24, 135.23, 135.22, 135.21, 135.19), (134.48, 134.46, 134.43, 134.40, 134.38), 128.98, 128.40, (123.03, 122.93, 122.83), (120.38, 120.37, 120.34, 120.32), 114.66, (54.10, 54.08, 54.06, 54.05, 54.03), (44.17, 44.16, 44.14, 44.09, 44.07, 44.00, 43.98, 43.96, 43.88, 43.77, 43.76, 43.68, 43.63, 43.58, 43.55, 43.49, 43.46, 43.38), (40.06, 40.05, 40.03), (39.25, 39.23, 39.22, 39.19), (35.09, 35.05, 35.01, 34.98, 34.97), (34.24, 34.23, 34.20, 34.19, 34.16, 34.11, 34.09, 34.08, 34.03, 34.02, 33.99, 33.98, 33.91, 33.89, 33.87, 33.86), 32.70, 29.71, (28.88, 28.87, 28.85, 28.84), (28.56, 28.48, 28.47, 28.46, 28.44), (28.21, 28.18, 28.15, 28.14, 28.12), (27.29, 27.27, 27.24, 27.23, 27.16, 27.08, 27.05), (26.01, 25.98, 25.98, 25.96), 23.01, (22.88, 22.83, 22.82, 22.80), (14.21, 14.16), 14.05, (10.95, 10.93, 10.92, 10.90, 10.88, 10.87), 10.64, 10.50, (10.41, 10.39, 10.34, 10.31). HRMS (MALDI+ FT-ICR): m/z $[M]^+$ calcld for $[C_{76}H_{108}N_2O_2S_6]^+$ 1272.67296, found 1272.67170 (err = 1.0 ppm).

IDTEH-7B[7]**.** A 15 ml high-pressure reaction tube (15 mL) containing **9** (0.284 g, 0.273 mmol, 1.0 equiv) and **7B** (0.221 g, 0.61 mmol, 2.24 equiv) and freshly distilled toluene (12 mL) was purged with N_2 for 20 min. Following the addition of $Pd(PPh_3)_4$ (0.0157 g, 0.014 mmol, 5 mol%) as a catalyst, the pressure tube was tightly closed and stirred in the oil bath at 110 °C. Incremental amounts of the catalyst (5 mol% each) were introduced at the 16th and the 21st h. After completion of the reaction in 36 h, toluene was removed on rotavapor, followed by extraction of the residue with DCM. The collected fractions were dried with brine and $MgSo_4$, and concentrated under rotary evaporator. Finally, the crude mixture was passed through silica gel (200-300 mesh size) by flash column chromatography, using EtOAc/$CHCl_3$ (0/100 to 5/95) solvent system, to give red powder IDTEH7B (139 mg, 0.11 mmol, 40%) with R_f = 0.58 (TLC (SiO_2), EtOAc/DCM: 5/95, blue-colored round spot).

Analytical data for **IDTEH-7B:** red powder; ^{1}H NMR (400 MHz, $CDCl_3$) δ 10.37 (s, 1H), 7.39 (s, 1H), 7.32 (t, J = 2.4 Hz, 1H), 3.06 (d, J = 7.0 Hz, 2H), 2.00 (dp, J = 12.9, 6.2, 5.1 Hz, 6H), 1.46 (dt, J = 19.5, 6.8 Hz, 2H), 1.39 – 1.33 (m, 4H), 1.01 – 0.89 (m, 14H), 0.70 (t, J = 6.8 Hz, 11H), 0.65 – 0.45 (m, 14H). ^{13}C{^{1}H} NMR (101 MHz, $CDCl_3$) δ 183.73, 180.90, 163.68, (156.86, 156.79, 156.76, 156.71, 156.71, 156.66, 156.62), (153.65, 153.62), 145.00, 136.49, 135.88, (133.78, 133.74), (129.99, 129.93), 122.97, (121.96, 121.89, 121.81, 121.74, 121.67, 121.60), 114.63, (54.38, 54.34, 54.31), (44.17, 44.07, 44.01, 43.94, 43.90, 43.82, 43.73, 43.62, 43.56, 43.48, 43.48, 43.39), 40.32, 39.98, (35.14, 35.09), (34.26, 34.24, 34.17, 34.00, 33.89, 33.76), 32.73, 31.95, 29.72, (28.79, 28.69, 28.57), (28.24, 28.20), (27.37, 27.26, 27.11), 25.99, (22.90, 22.88, 22.80, 22.70), (14.16, 14.11, 14.07, 14.04), (10.80, 10.72), 10.57, (10.40,

10.35). HRMS. (MALDI+ FT-ICR): *m/z* $[M]^+$ calcld for $[C_{76}H_{108}N_2O_2S_6^+$ 1272.67295, found 1272.67188 (err = -0.8 ppm).

IDTEH-7A-2FIC[7, 8]**.** Knoevenagel condensation was carried out using a mixture of IDTEH-7A (42.50 mg, 0.033 mmol, 1.0 equiv), 2FIC (22.78 mg, 0.099 mmol, 3.0 equiv) and EtOH (15 mL) in a high-pressure reaction tube (38 mL). After purging the reaction mixture with N_2 for 20 min, the reaction tube was tightly closed and stirred at 100 °C. The reaction tube was transferred to ambient temperature (22 °C) after 12 h, followed by filtration of the resulting mixture. The filter residue was subsequently washed with EtOH, MeOH, and *n*-Hexane. The crude solid was loaded onto the silica-gel (200-300 mesh size), followed by elution with DCM/Hex (08/02) solvent system. The product was further purified by precipitation in DCM:MeOH mixture to obtain dark green solid IDTEH-7A-2FIC (41.2 mg, 0.024 mmol, 73%) with R_f = 0.85 (TLC (SiO_2), DCM, green-colored round spot).

Analytical data for **IDTEH-7A-2FIC:** dark green solid; ^1H NMR (700 MHz, $CDCl_3$) δ 8.90 (s, 2H), 8.52 (dd, *J* = 9.8, 6.4 Hz, 2H), 7.99 – 7.93 (m, 2H), 7.67 (t, *J* = 7.3 Hz, 2H), 7.49 (s, 2H), 3.13 – 3.10 (m, 4H), 2.06 (dd, *J* = 13.6, 5.3 Hz, 8H), 2.00 (overlapped m, 4H), 1.53 – 1.45 (m, 8H), 1.42 – 1.35 (m, 8H), 0.98 (dt, *J* = 24.6, 6.7 Hz, 34H), 0.71 (t, *J* = 6.8 Hz, 18H), 0.65 – 0.52 (m, 18H). $^1H\{^{19}F\}$ NMR (700 MHz, $CDCl_3$) δ 8.90 (s, 2H), 8.53 (s, 2H), 7.98 – 7.93 (m, 2H), 7.67 (s, 2H), 7.49 (s, 2H), 3.13 – 3.09 (m, 4H), 2.09 – 2.04 (m, 8H), 2.02 (overlapped dt, *J* = 12.6, 6.0 Hz, 4H), 1.49 (dt, *J* = 21.7, 6.7 Hz, 8H), 1.42 – 1.35 (m, 8H), 0.98 (dt, *J* = 25.6, 7.0 Hz, 34H), 0.70 (d, *J* = 7.5 Hz, 18H), 0.65 – 0.51 (m, 18H). $^{13}C\{^1H\}$ NMR (176 MHz, $CDCl_3$) δ 186.64, (178.14, 178.07, 178.01), (157.99, 157.97, 157.92, 157.85, 157.83, 157.78, 157.76), 157.67, 154.87, 153.86, 153.47 (dd, *J* = 23.80, 13.30 Hz), 150.75, (142.85, 142.80, 142.77), 136.82, (136.73, 136.69), (136.13, 136.11), 134.26, 134.03, (125.60, 125.52, 125.50, 125.46, 125.37), 119.74, 118.53, 115.42, 115.07, 114.91 (d, *J* = 21.40 Hz), 114.75, 112.26 (d, *J* = 18.30 Hz), 67.54, 54.20, (44.16, 44.11, 44.05, 44.01, 43.99, 43.91, 43.82, 43.68, 43.58), 40.19, (39.52, 39.50), (35.20, 35.16, 35.10), (34.35, 34.31, 34.23, 34.20, 33.83, 33.81, 33.71, 33.69), 32.61, 29.71, 28.76, (28.57, 28.49, 28.47), (28.12, 28.09), (27.40, 27.25, 27.08, 26.89, 26.87), (25.97, 25.95, 25.92, 25.90), 23.05, (22.88, 22.83, 22.79), (14.21, 14.16), 14.03, (10.87, 10.82), 10.65, 10.54, (10.31, 10.25). (No peak was observed for one of the two carbon atoms, attached directly to F.) $^{13}C\{^{19}F, ^1H\}$ NMR (176 MHz, $CDCl_3$) δ 186.65, (178.15, 178.08, 178.02), (157.93, 157.86, 157.80), 157.68, 154.89, 154.29, 154.16, 153.89, 150.78, (142.82, 142.78), 136.84, 136.72, 136.14, 134.26, 134.04, (125.64, 125.61, 125.54, 125.50, 125.36, 125.30), 119.76, 118.54, 115.43, 115.08, 114.92, 114.77, 112.27, 67.55, 54.21, (44.18, 44.12, 44.01, 43.96, 43.85, 43.79, 43.69), 40.20, 39.50, (35.21, 35.16, 35.11), (34.42, 34.35, 34.32, 34.24, 34.21, 33.82, 33.70), 32.62, 29.72, 28.77, (28.57, 28.48), (28.13, 28.10), (27.41, 27.26, 27.08, 26.89), 25.95, 23.06, (22.89, 22.81), (14.22, 14.17), 14.04, (10.87, 10.83), 10.66, 10.55, (10.31, 10.26). ^{19}F NMR (659 MHz, $CDCl_3$) δ -123.54 – -123.61 (m, 2F), -124.91 – -124.98 (m, 2F). $^{19}F\{^1H\}$ NMR (659 MHz, $CDCl_3$) δ -123.58 (dq, *J* = 20.7, 13.4, 10.7 Hz, 2F), -124.95 (dt, *J* = 17.4, 7.4 Hz, 2F). HRMS (MALDI+ FT-ICR): *m/z* $[M]^+$ calcld for $[C_{100}H_{112}F_4N_6O_2S_6]^+$ 1696.71016, found 1696.71062 (err = -0.3 ppm).

IDTEH-7B-2FIC. Using IDTEH-7B (70.00 mg, 0.054 mmol, 1.0 equiv) as limiting reactant, Knoevenagel condensation was carried out under similar conditions as previously described for the synthesis of IDTEH-7A-2FIC. The flash chromatography was run using DCM as eluant and the product was further purified by precipitation in DCM:MeOH mixture to obtain dark green solid IDTEH-7B-2FIC (52.8 mg, 0.031 mmol, 57%) with R_f = 0.70 (TLC (SiO_2), DCM, green-colored round spot).

Analytical data for **IDTEH-7B-2FIC:** dark green solid; ^1H NMR (700 MHz, $CDCl_3$) δ 9.70 (s, 2H), 8.57 – 8.53 (m, 2H), 7.68 (t, *J* = 7.3 Hz, 2H), 7.64 (s, 2H), 7.47 (s, 2H), 3.16 – 3.11 (m, 4H), 2.06 (dd, *J* = 10.9, 5.9 Hz, 8H), 2.02 (dd, *J* = 12.6, 6.4 Hz, 4H), 1.53 – 1.48 (m,

4H), 1.48 – 1.40 (m, 4H), 1.35 (dq, J = 14.2, 7.3 Hz, 8H), 0.99 (t, J = 7.4 Hz, 17H), 0.93 (t, J = 7.0 Hz, 17H), 0.73 – 0.69 (m, 18H), 0.66 – 0.53 (m, 18H). $^{1}H\{^{19}F\}$ NMR (700 MHz, $CDCl_3$) δ 9.70 (s, 2H), 8.54 (s, 2H), 7.68 (s, 2H), 7.64 – 7.63 (m, 2H), 7.46 (s, 2H), 3.14 (dd, J = 6.4, 4.5 Hz, 4H), 2.04 (overlapped m, 12H), 1.53 – 1.48 (m, 4H), 1.50 – 1.39 (m, 4H), 1.39 – 1.32 (m, 8H), 0.98 (t, J = 7.4 Hz, 17H), 0.93 (t, J = 6.9 Hz, 17H), 0.73 – 0.67 (m, 18H), 0.66 – 0.51 (m, 18H). $^{13}C\{^{1}H\}$ NMR (176 MHz, $CDCl_3$) δ 186.69, 184.30, (169.98, 169.94, 169.89), (158.24, 158.15, 158.11), 158.02, 154.96 (dd, J = 25.20, 14.40 Hz), 154.42, 153.47 (dd, J = 28.50, 14.10 Hz), 148.07, (141.06, 141.01), 137.27, 136.86, 136.36, 134.43, 133.75, (129.84, 129.81), (123.74, 123.66, 123.58, 123.51, 123.43, 123.36), 120.95, 118.98, 115.17, 114.91 (d, J = 16.70 Hz), 114.26, 112.28 (d, J = 17.70 Hz), 68.92, (54.51, 54.45, 54.41, 54.34), (44.25, 44.13, 44.10, 44.03, 43.96, 43.90, 43.80, 43.75, 43.66, 43.57), 40.34, 39.84, (35.22, 35.18, 35.12), (34.35, 34.31, 34.25, 34.22), (33.75, 33.65, 33.63), 32.78, 29.72, 28.85, (28.65, 28.55), 28.10, (27.45, 27.30, 27.06, 26.89), 25.96, (22.89, 22.80), (14.20, 14.11, 14.05), (10.85, 10.73, 10.60), (10.31, 10.27). $^{13}C\{^{19}F, ^{1}H\}$ NMR (176 MHz, $CDCl_3$) δ 186.68, 184.30, (169.97, 169.93, 169.89), (158.23, 158.15, 158.11), 158.0-0, 154.42, 154.29, 154.13, 148.07, 141.05, 137.26, 136.87, 136.36, 134.43, 133.74, (129.96, 129.92, 129.87, 129.84, 129.81), (123.74, 123.66, 123.59, 123.51, 123.44, 123.41, 123.31, 123.28), 120.95, 118.97, 115.17, 114.89, 114.25, 112.27, 68.92, 54.44, (44.25, 44.14, 44.10, 44.02, 43.96, 43.90, 43.79, 43.76, 43.67, 43.60, 43.55), 40.34, 39.83, (35.32, 35.22, 35.18, 35.13), (34.34, 34.30, 34.24, 34.15), (33.76, 33.65, 33.63), 32.78, 29.72, 28.85, (28.65, 28.55), 28.10, (27.45, 27.30, 27.06, 26.89), 25.96, (22.89, 22.80), (14.19, 14.11, 14.05), (10.85, 10.72, 10.60), (10.31, 10.27). ^{19}F NMR (659 MHz, $CDCl_3$) δ -123.48 – -123.58 (m, 2F), -125.02 – -125.10 (m, 2F). $^{19}F\{^{1}H\}$ NMR (659 MHz, $CDCl_3$) δ -123.53 (dt, J = 19.4, 10.6 Hz, 2F), -125.06 (d, J = 19.3 Hz, 2F). HRMS (MALDI+ FT-ICR): *m/z* [M]s^{+} calcld for $[C_{100}H_{112}F_4N_6O_2S_6]^{+}$ 1696.71016, found 1696.71032 (err = 0.1 ppm).

Absorption and Emission Studies

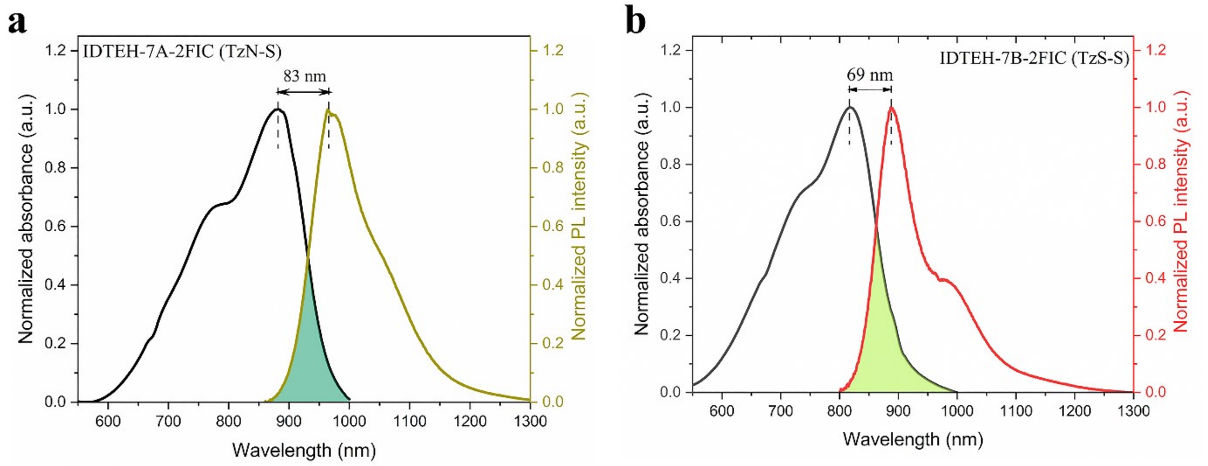

Figure S1. Stokes shift obtained from emission and absorption spectra of TzN-S (a) and TS-S (b) in thin film state.

Electrochemical Properties

Table S1. Electrochmical parameters, extracted from voltammograms of the materials understudy.

Film	Oxidation[a)]		Reduction[a)]					
	$E_{ox}^{1/2}$ [V][b)]	E_{ox}^{onset} [V]	E_{red} [V]		E_{red}^{onset} [V]	E_{HOMO}^{CV} [eV][c)]	E_{LUMO}^{CV} [eV][d)]	E_{g}^{CV} [eV][e)]
**TzN-S**	1.19	1.11	E_{Pa}	-	− 0.42	− 5.49	− 3.96	1.53
			E_{Pc}	− 0.51				
**TzS-S**	1.24	1.13	E_{Pa}	-	− 0.52	− 5.51	− 3.86	1.66
			E_{Pc}	− 0.57				

All measurements were taken at r.t. [a)][vs Ag/AgCl]; [b)][$E_{ox}^{1/2} = (E_{Pa} + E_{Pc})/2$] has been used to calculate the half-wave potential;[27] the half-wave potential ($E^{1/2}$) of ferrocene couple (FC/FC^+), was found at 0.42 V vs Ag/AgCl and we used the following expressions[9] to estimate the energy levels and electrochemical band gap:
[c)]$E_{HOMO}^{CV} = -e[E_{ox}^{onset} - E^{1/2}(FC/FC^+) + 4.8]$; [d)]$E_{LUMO}^{CV} = -e[E_{red}^{onset} - E^{1/2}(FC/FC^+) + 4.8]$; [e)]$E_{g}^{CV} = E_{LUMO}^{CV} - E_{HOMO}^{CV}$

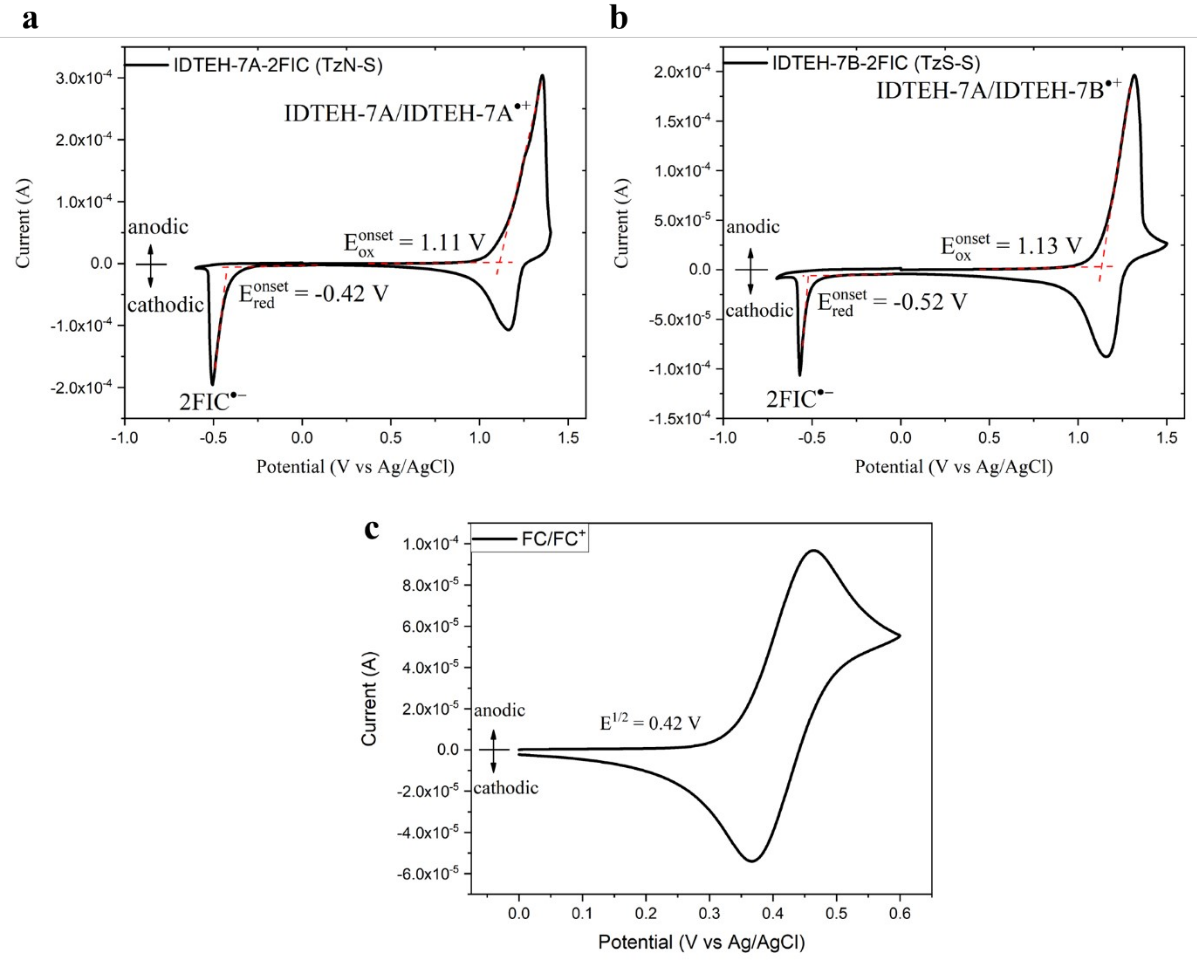

Figure S2. Cyclic voltammograms of synthesized NFA acceptors and Ferrocene

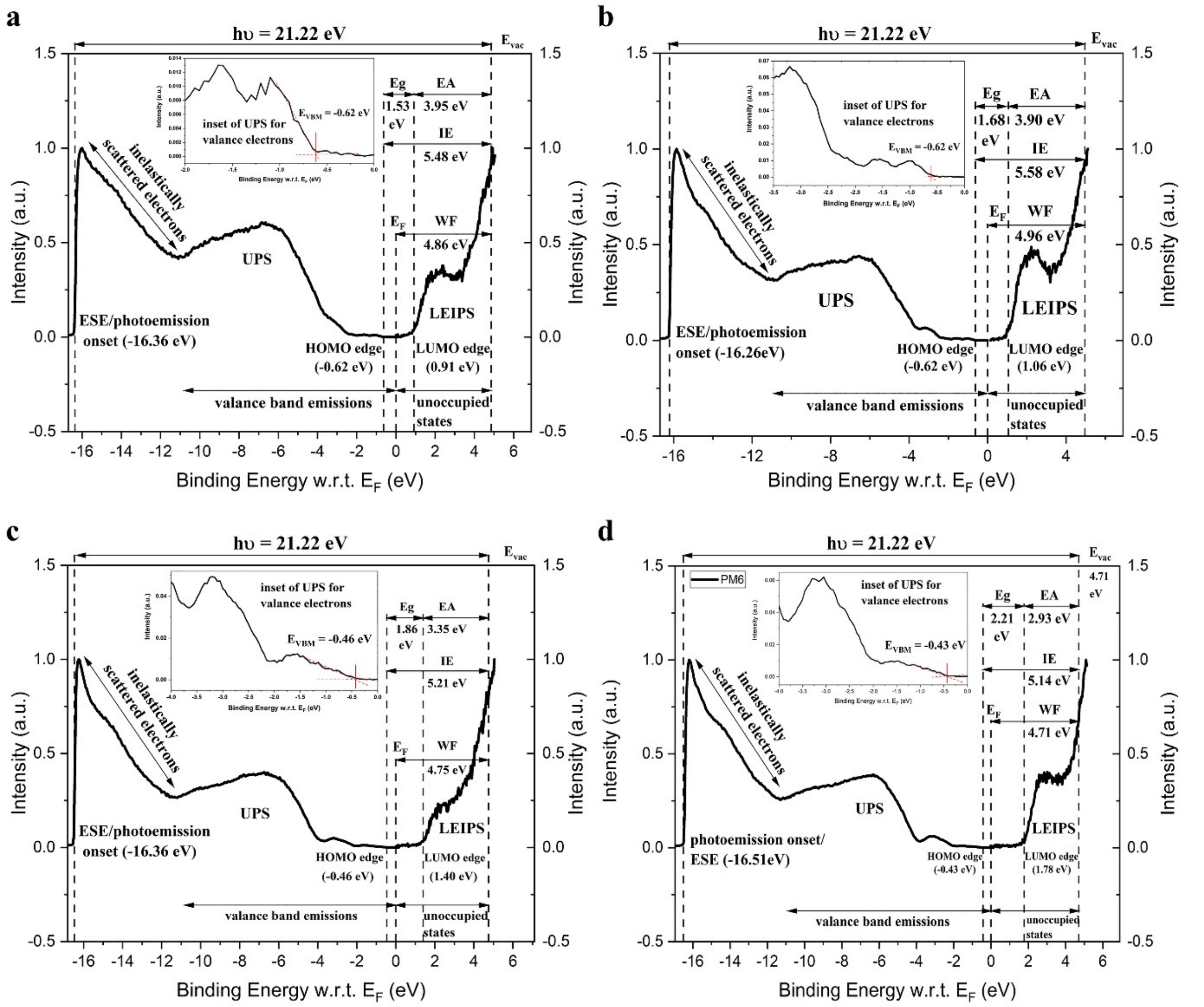

Figure S3. Combined UPS and LE-IPES spectra obtained from ~15 nm thick films of **a)** IDTEH-7A-2FIC; **b)** IDTEH-7B-2FIC; **c)** PCE10; and **d)** PM6, deposited on ITO-coated glass substrates. The given electronic energies have been derived while referenced to the vacuum level (E_{vac}) using the expressions:[10, 11] ionization energy $(IE) = h\nu - (ESE - E_{VBM})$ and $WF = h\nu - ESE$; electron affinity (EA) has been determined directly from LE-IPES data.

Table S2. Photovoltaic performance of OSC devices[a] with different D:A blend ratio

D:A (w/w)	V_{oc} [V]	J_{sc} [mA cm^{-2}]	FF	PCE [%]
1.0:1.0	0.74	21.83	0.61	9.89
	(0.74 ± 0.01)	(21.42 ± 0.36)	(0.59 ± 0.03)	(9.31 ± 0.61)
1.0:1.3	0.74	21.52	0.64	10.22
	(0.74 ± 0.00)	(21.31 ± 0.15)	(0.63 ± 0.01)	(9.95 ± 0.19)
1.0:1.5[b]	0.75	22.09	0.65	10.75
	(0.75 ± 0.00)	(21.36 ± 0.36)	(0.66 ± 0.00)	(10.44 ± 0.16)
1.0:2.0	0.74	21.39	0.64	10.02
	(0.74 ± 0.00)	(21.31 ± 0.11)	(0.63 ± 0.00)	(9.90 ± 0.09)

[a]Conventional architecture: ITO/PEDOT:PSS/PCE10:**TzS-S**/PDINN/Ag; The average values and standard deviations in parentheses, represent the statistical data obtained from eight and [b]eighteen independent cells.

Table S3. Photovoltaic performance of OSC devices[a] with different D:A blend ratio

D:A (w/w)	V_{oc} [V]	J_{sc} [mA cm^{-2}]	FF	PCE [%]
1.0:1.0	0.71	13.38	0.57	05.40
	(0.71 ± 0.04)	(13.18 ± 0.26)	(0.56 ± 0.03)	(5.21 ± 0.17)
1.0:1.3	0.70	14.12	0.58	05.72
	(0.70 ± 0.00)	(14.10 ± 0.15)	(0.57 ± 0.00)	(5.69 ± 0.02)
1.0:1.5[b]	0.70	14.27	0.61	06.13
	(0.70 ± 0.00)	(13.23 ± 0.50	(0.63 ± 0.016)	(5.80 ± 0.22)
1.0:2.0	0.70	13.44	0.63	5.92
	(0.70 ± 0.00)	(13.58 ± 0.17)	(0.62 ± 0.01)	(5.88 ± 0.03)

[a]Conventional architecture: ITO/PEDOT:PSS/PCE10:**TzN-S**/PDINN/Ag; The average values and standard deviations in parentheses, represent the statistical data obtained from eight and [b]eighteen independent cells.

Photovoltaic Parameters

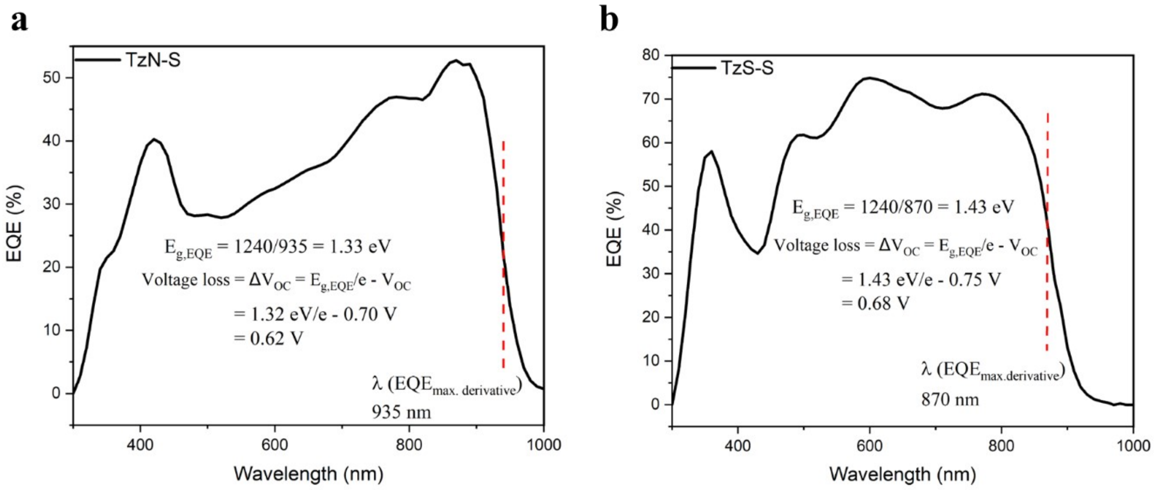

Figure S4. Voltage loss estimation for TzN-S and TzS-S based OPV devices. $E_{g,pv}$ or $E_{g,EQE}$ is extracted from the maximum of the derivative of EQE_{PV}, representing an analogy to ideal Shockley-Queisser bandgap for real photovoltaic device.[12, 13]

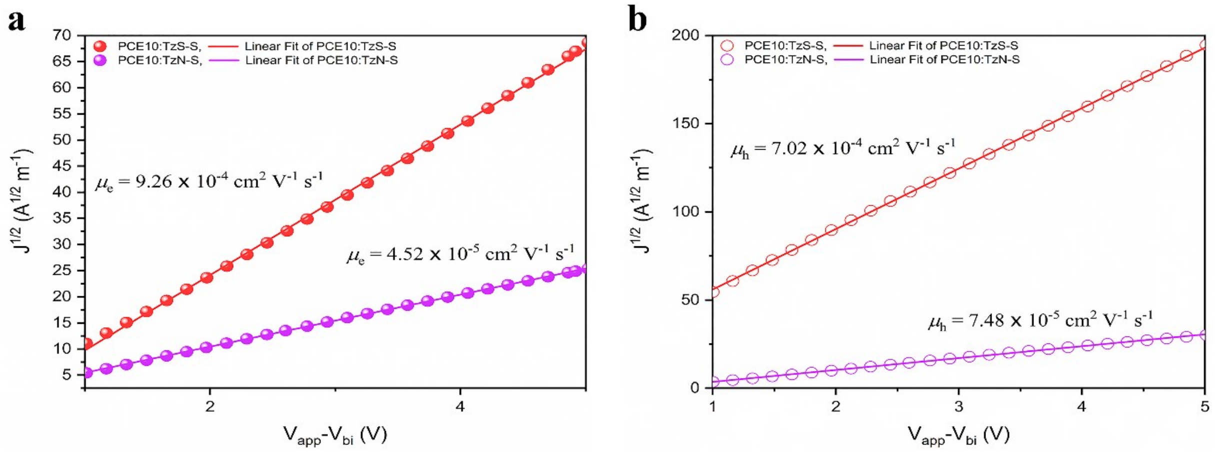

Figure S5. charge carrier mobility measured from electron only (a) and hole only (b) devices.

Table S4. Photovoltaic performance of OSC devices[a] under varying annealing conditions

D:A (w/w)	Annealing	Treatment	V_{oc} [V]	J_{sc} [mA cm^{-2}]	FF	PCE [%]
1.0:1.5	as-cast		0.75<br>(0.75 ± 0.00)	22.09<br>(21.36 ± 0.36)	0.65<br>(0.66 ± 0.00)	10.75[b]<br>(10.44 ± 0.16)
		80 °C, 10 min	0.74<br>(0.74 ± 0.00)	21.91<br>(21.76 ± 0.20)	0.64<br>(0.63 ± 0.02)	10.38<br>(10.15 ± 0.32)
		110 °C, 10 min	0.74<br>(0.74 ± 0.00)	21.86<br>(21.57 ± 0.32)	0.63<br>(0.62 ± 0.02)	10.20<br>(9.80 ± 0.48)
	SVA	CF, 15 s	0.74<br>(0.73 ± 0.00)	21.81<br>(21.53 ± 0.18)	0.63<br>(0.63 ± 0.00)	10.15<br>(9.99 ± 0.14)

[a]Conventional architecture: ITO/PEDOT:PSS/PCE10:**TzS-S**/PDINN/Ag; The average values and standard deviations in parentheses, represent the statistical data obtained from eight and [b]eighteen independent cells.

Table S5. Photovoltaic performance of OSC devices[a] under varying annealing conditions

D:A (w/w)	Annealing	Treatment	V_{oc} [V]	J_{sc} [mA cm^{-2}]	FF	PCE [%]
1.0:1.5	as-cast		0.70<br>(0.70 ± 0.00)	14.27<br>(13.23 ± 0.51)	0.61<br>(0.63 ± 0.02)	06.13[b]<br>(5.80 ± 0.22)
	TA	80 °C, 10 min	0.70<br>(0.70 ± 0.00)	13.47<br>(12.91 ± 0.50)	0.64<br>(0.63 ± 0.02)	06.00<br>(5.62 ± 0.22)
		110 °C, 10 min	0.70<br>(0.70 ± 0.00)	14.42<br>(13.64 ± 0.40)	0.60<br>(0.60 ± 0.03)	06.07<br>(5.72 ± 0.37)
	SVA	CF, 15 s	0.70<br>(0.70 ± 0.00)	13.74<br>(13.40 ± 0.37)	0.62<br>(0.61 ± 0.02)	05.96<br>(5.70 ± 0.2)

[a]Conventional architecture: ITO/PEDOT:PSS/PCE10:**TzN-S**/PDINN/Ag; The average values and standard deviations in parentheses, represent the statistical data obtained from eight and [b]eighteen independent cells.

Table S6. Photovoltaic performance of OSC devices[a] with different additives under different annealing conditions

D:A (w/w)	Annealing	Treatment	Additive	V_{oc} [V]	J_{sc} [mA cm^{-2}]	FF	PCE [%]
1.0:1.5	as-cast		W/O	0.75<br>(0.75 ± 0.00)	22.09<br>(21.36 ± 0.36)	0.65<br>(0.66 ± 0.00)	10.75[b]<br>(10.44 ± 0.16)
			W/CN	0.73<br>(0.73 ± 0.00)	21.49<br>(21.22± 0.17)	0.62<br>(0.61± 0.01)	9.73<br>(9.50 ± 0.13)
			W/DIO	0.73<br>(0.72 ± 0.00)	19.78<br>(18.70 ± 1.58)	0.47<br>(0.46 ± 0.02)	6.84<br>(6.32 ± 0.75)
	TA	80 °C, 10 min	W/O	0.74<br>(0.74 ± 0.00)	21.91<br>(21.76 ± 0.12)	0.64<br>(0.63 ± 0.02)	10.38<br>(10.15 ± 0.32)
			W/CN	0.74<br>(0.73 ± 0.00)	21.56<br>(21.35 ± 0.16)	0.62<br>(0.61 ± 0.01)	9.88<br>(9.55 ± 0.24)
			W/DIO	0.73<br>(0.72 ± 0.00)	20.47<br>(20.27 ± 0.01)	0.49<br>(0.49 ± 0.00)	7.27<br>(7.17 ± 0.08)
		110 °C, 10 min	W/O	0.74<br>(0.74 ± 0.00)	21.86<br>(21.57 ± 0.32)	0.63<br>(0.62 ± 0.02)	10.20<br>(9.80 ± 0.48)
			W/CN	0.73<br>(0.73 ± 0.00)	21.60<br>(21.34 ± 0.27)	0.61<br>(0.60 ± 0.00)	9.57<br>(9.33 ± 0.18)
			W/DIO	0.72<br>(0.72 ± 0.00)	22.19<br>(20.90 ± 0.58)	0.49<br>(0.49 ± 0.01)	7.82<br>(7.40 ± 0.18)
	SVA	CF, 15 s	W/O	0.74<br>(0.73 ± 0.00)	21.81<br>(21.53 ± 0.18)	0.63<br>(0.63 ± 0.00)	10.15<br>(9.99 ± 0.14)
			W/CN	0.73<br>(0.72 ± 0.01)	21.08<br>(19.59 ± 2.23)	0.61<br>(0.53 ± 0.06)	9.36<br>(7.56 ± 1.68)
			W/DIO	0.67<br>(0.67 ± 0.01)	14.26<br>(12.89 ± 1.36)	0.47<br>(0.46 ± 0.01)	4.53<br>(3.97 ± 0.57)

[a]Conventional architecture: ITO/PEDOT:PSS/PCE10:**TzS-S**/PDINN/Ag; The average values and standard deviations in parentheses, represent the statistical data obtained from eight and [b]eighteen independent cells.

Table S7. Effect of varying concentration of additives on photovoltaic performance of devices[a]

D:A (w/w)	Annealing	Treatment	Additive[x]	V_{oc} [V]	J_{sc} [mA cm^{-2}]	FF	PCE [%]
1.0:1.5	as-cast		W/CN[0.25]	0.72 (0.72 ± 0.00)	18.55 (18.50 ± 0.06)	0.51 (0.51 ± 0.00)	06.82 (6.76 ± 0.06)
			W/CN[0.50]	0.73 (0.73 ± 0.00)	21.49 (21.22± 0.17)	0.62 (0.61± 0.01)	09.73 (9.50 ± 0.13)
			W/CN[1.0]	0.66 (0.65 ± 0.00)	14.81 (14.86 ± 0.05)	0.48 (0.48 ± 0.00)	04.67 (4.64 ± 0.03)
	TA	110 °C, 10 min	W/CN[0.25]	0.73 (0.72 ± 0.00)	18.43 (18.24 ± 0.14)	0.50 (0.49 ± 0.01)	06.64 (6.41 ± 0.18)
			W/CN[0.50]	0.74 (0.74 ± 0.00)	21.86 (21.57 ± 0.32)	0.63 (0.62 ± 0.02)	10.20 (9.80 ± 0.48)
			W/CN[1.0]	0.68 (0.66 ± 0.01)	14.39 (14.64 ± 0.17)	0.56 (0.50 ± 0.03)	5.55 (4.80 ± 0.36)

[a]Conventional architecture: ITO/PEDOT:PSS/PCE10:**TzS-S**/PDINN/Ag; The average values and standard deviations in parentheses, represent the statistical data obtained from eight independent cells.

Table S8. Photovoltaic performance of OSC devices[a] with different additives under different annealing conditions

D:A (w/w)	Annealing	Treatment	Additive	V_{oc} [V]	J_{sc} [mA cm^{-2}]	FF	PCE [%]
1.0:1.5	as-cast		W/O	0.70 (0.70 ± 0.00)	14.27 (13.23 ± 0.51)	0.61 (0.63 ± 0.02)	6.13[b] (5.80 ± 0.22)
			W/CN	0.71 (0.71 ± 0.00)	14.22 (13.84 ± 0.26)	0.58 (0.58 ± 0.00)	5.87 (5.71 ± 0.10)
			W/DIO	0.69 (0.68 ± 0.00)	11.64 (10.12 ± 0.67)	0.61 (0.58 ± 0.05)	04.87 (4.04 ± 0.46)
	TA	80 °C, 10 min	W/O	0.70 (0.70 ± 0.00)	13.47 (12.91 ± 0.50)	0.64 (0.63 ± 0.02)	6.00 (5.62 ± 0.22)
			W/CN	0.70 (0.69 ± 0.00)	12.94 (11.70 ± 0.72)	0.63 (0.64 ± 0.01)	5.74 (5.21 ± 0.31)
			W/DIO	0.69 (0.69 ± 0.00)	10.85 (10.14 ± 0.37)	0.59 (0.59 ± 0.01)	04.39 (4.11 ± 0.16)
		110 °C, 10 min	W/O	0.70 (0.70 ± 0.00)	14.42 (13.64 ± 0.40)	0.60 (0.60 ± 0.03)	6.07 (5.72 ± 0.37)
			W/CN	0.71 (0.70 ± 0.00)	13.95 (13.47 ± 0.33)	0.58 (0.57 ± 0.01)	5.73 (5.45 ± 0.18)
			W/DIO	0.69 (0.68 ± 0.00)	10.11 (9.71 ± 0.25)	0.58 (0.59 ± 0.01)	04.03 (3.91 ± 0.08)
	SVA	CF, 15 s	W/O	0.70 (0.70 ± 0.00)	13.74 (13.40 ± 0.37)	0.62 (0.61 ± 0.02)	05.96 (5.70 ± 0.23)
			W/CN	0.71 (0.70 ± 0.01)	14.36 (13.14 ± 1.14)	0.58 (0.55 ± 0.03)	5.92 (5.02 ± 0.80)
			W/DIO	0.68 (0.68 ± 0.00)	10.21 (9.81 ± 0.24)	0.58 (0.58 ± 0.02)	04.07 (3.88 ± 0.16)

[a]Conventional architecture: ITO/PEDOT:PSS/PCE10:**TzN-S**/PDINN/Ag; The average values and standard deviations in parentheses, represent the statistical data obtained from eight and [b]eighteen independent cells.

Table S9. Photovoltaic performance of devices[a] with ZnO NPs (22 mg/mL) as ETL

D:A (w/w)	Annealing	Treatment	Additive	V_{oc} [V]	J_{sc} [mA cm^{-2}]	FF	PCE [%]
1.0:1.5	as-cast		W/O	0.69 (0.65 ± 0.04)	22.82 (21.20 ± 0.84)	0.46 (0.41 ± 0.03)	7.08 (5.71 ± 0.80)
			W/CN	0.70 (0.56 ± 0.21)	20.96 (20.16 ± 0.72)	0.47 (0.39 ± 0.07)	6.86 (4.79 ± 2.16)
	TA	80 °C, 10 min	W/O	0.59 (0.51 ± 0.06)	21.89 (21.21 ± 0.32)	0.41 (0.39 ± 0.02)	5.36 (4.24 ± 0.72)
			W/CN	0.62 (0.58 ± 0.05)	19.90 (19.86 ± 0.04)	0.41 (0.38 ± 0.03)	5.13 (4.45 ± 0.68)
		110 °C, 10 min	W/O	0.60 (0.55 ± 0.03)	21.79 (21.59 ± 0.56)	0.39 (0.38 ± 0.01)	5.13 (4.49 ± 0.36)
			W/CN	0.61 (0.50 ± 0.11)	20.76 (19.84 ± 1.01)	0.39 (0.37 ± 0.02)	4.93 (3.73 ± 1.15)

[a]Inverted architecture: ITO/ZnO NPs/PCE10:**TzS-S**/MoO_3/Ag; The average values and standard deviations in parentheses, represent the statistical data obtained from eight independent cells.

Table S10. Photovoltaic performance of devices[a] with ZnO NPs (22 mg/mL) as ETL

D:A (w/w)	Annealing	Treatment	Additive	V_{oc} [V]	J_{sc} [mA cm^{-2}]	FF	PCE [%]
1.0:1.5	as-cast		W/O	0.43	14.49	0.30	1.90
				(0.40 ± 0.05)	(13.68 ± 0.60)	(0.30 ± 0.01)	(1.65 ± 0.22)
			W/CN	0.67	12.42	0.36	2.96
				(0.53 ± 0.11)	(13.01 ± 0.70)	(0.35 ± 0.02)	(2.37 ± 0.40)
	TA	80 °C, 10 min	W/O	0.70	17.31	0.52	6.30
				(0.64 ± 0.11)	(15.97 ± 0.74)	(0.47 ± 0.06)	(4.90 ± 1.23)
			W/CN	0.69	15.52	0.49	5.30
				(0.69 ± 0.00)	(15.34 ± 0.18)	(0.50 ± 0.00)	(5.23 ± 0..06)
		110 °C, 10 min	W/O	0.70	14.68	0.51	5.25
				(0.67 ± 0.03)	(15.32 ± 0.87)	(0.44 ± 0.04)	(4.57 ± 0.47)
			W/CN	0.68	17.00	0.40	4.54
				(0.63 ± 0.04)	(15.18 ± 1.17	(0.37 ± 0.02)	(3.54 ± 0.62)

[a]Inverted architecture: ITO/ZnO NPs/PCE10:**TzN-S**/MoO_3/Ag; The average values and standard deviations in parentheses, represent the statistical data obtained from eight independent cells.

Table S11. Photovoltaic performance of devices[a] with **PM6** as donor material

D:A (w/w)	Annealing	Treatment	Additive	V_{oc} [V]	J_{sc} [mA cm^{-2}]	FF	PCE [%]
1.0:1.5	as-cast		W/O	0.91	15.54	0.53	7.47
				(0.90 ± 0.01)	(15.42 ± 0.13)	(0.52 ± 0.00)	(7.35 ± 0.12)
			W/CN	0.88	16.51	0.55	8.06
				(0.89 ± 0.01)	(15.83 ± 0.49)	(0.53 ± 0.01)	(7.49 ± 0.33)
	TA	110 °C, 10 min	W/O	0.90	15.68	0.54	7.58
				(0.90 ± 0.00)	(15.59 ± 0.09)	(0.54 ± 0.00)	(7.54 ± 0.04)
			W/CN	0.88	16.42	0.54	7.81
				(0.87 ± 0.00)	(16.32 ± 0.09)	(0.54 ± 0.00)	(7.76 ± 0.04)

[a]Conventional architecture: ITO/PEDOT:PSS/PM6:**TzS-S**/PDINN/Ag; The average values and standard deviations in parentheses, represent the statistical data obtained from eight and [b]eighteen independent cells.

Table S12. Photovoltaic performance of devices[a] with **PM6** as donor material

D:A (w/w)	Annealing	Treatment	Additive	V_{oc} [V]	J_{sc} [mA cm^{-2}]	FF	PCE [%]
1.0:1.5	as-cast		W/O	0.72	16.42	0.64	7.57
				(0.71 ± 0.00)	(16.41 ± 0.01)	(0.64 ± 0.00)	(7.47 ± 0.09)
			W/CN	0.70	13.14	0.64	5.94
				(0.70 ± 0.00)	(13.08 ± 0.03)	(0.64 ± 0.00)	(5.91 ± 0.01)
	TA	110 °C, 10 min	W/O	0.71	12.38	0.62	5.38
				(0.70 ± 0.00)	(12.35 ± 0.15)	(0.61 ± 0.00)	(5.33 ± 0.10)
			W/CN	0.68	14.39	0.56	5.55
				(0.66 ± 0.01)	(14.59 ± 0.76)	(0.51 ± 0.03)	(4.90 ± 0.42)

[a]Conventional architecture: ITO/PEDOT:PSS/PM6:**TzN-S**/PDINN/Ag; The average values and standard deviations in parentheses, represent the statistical data obtained from eight independent cells.

Table S13. Charge carrier mobilities of newly synthesized regiomeric acceptor materials

Acceptors	Electron Mobility (μ_e)[a] [cm^2 V-1 s^{-1}]	Hole Mobility (μ_h)[b] [cm^2 V^{-1} s^{-1}]	Mobility Balance
TzN-S	4.52×10^{-5}	7.48×10^{-5}	1.65
TzS-S	9.26×10^{-4}	7.02×10^{-4}	1.32

[a]electron-only devices: ITO/ZnO Sg/PCE10:acceptor/PDINN/Ag

[b]hole-only devices: ITO/PEDOT:PSS/PCE10:acceptor/MoO_3/Ag

Table S14. V_{OC} and the calculated E_{loss} of devices prepared from PCE10 and two acceptors[a]

D:A (w/w)	BHJ	V_{oc} [V]	$E_{g, donor}$ [eV]	$E_{g, acceptor}$ [eV]	E_{loss} [eV]	$E_{g,EQE}$ [V]	ΔV_{oc} [V]
1.0:1.5	TzN-S	0.70	1.60	1.28	0.58	1.32	0.62
	TzS-S	0.75	1.60	1.37	0.62	1.43	0.68

[a]E_{loss} is defined as $E_{loss} = E_g - eV_{OC}$, where E_g is the lowest optical bandgap of the donor or acceptor component.

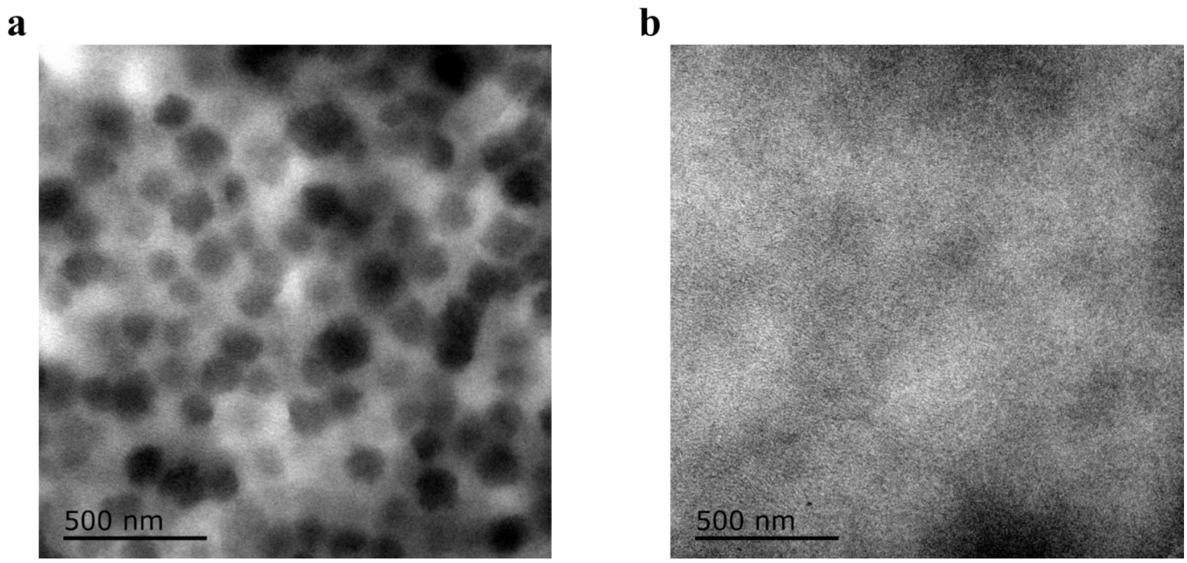

Figure S6. TEM images of **a**) PCE10:TzN-S and **b)** PCE10:TzS-S blends

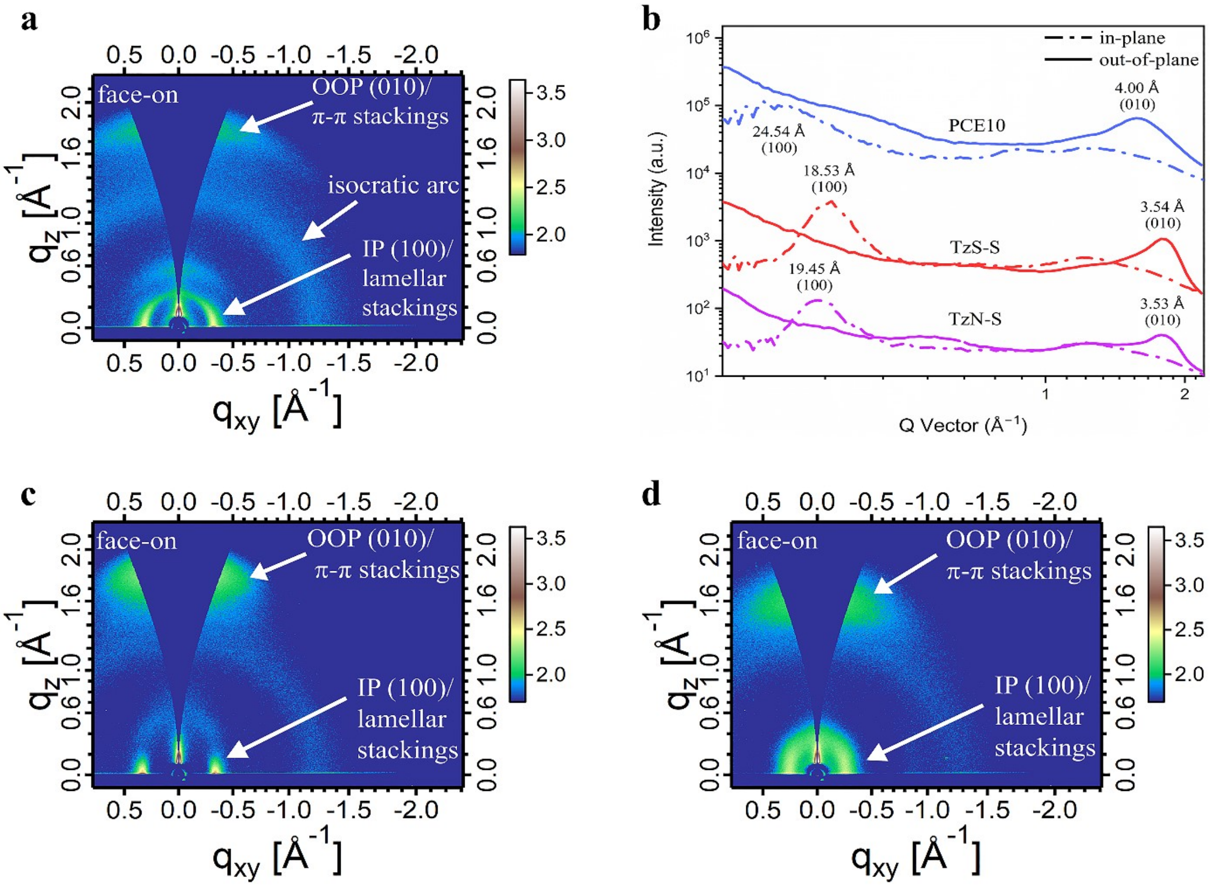

Figure S7. 2D GIWAXS images of pristine **a)** TzN-S, **b)** TzS-S and **c)** PCE10, and **d)** the corresponding line-cut profiles

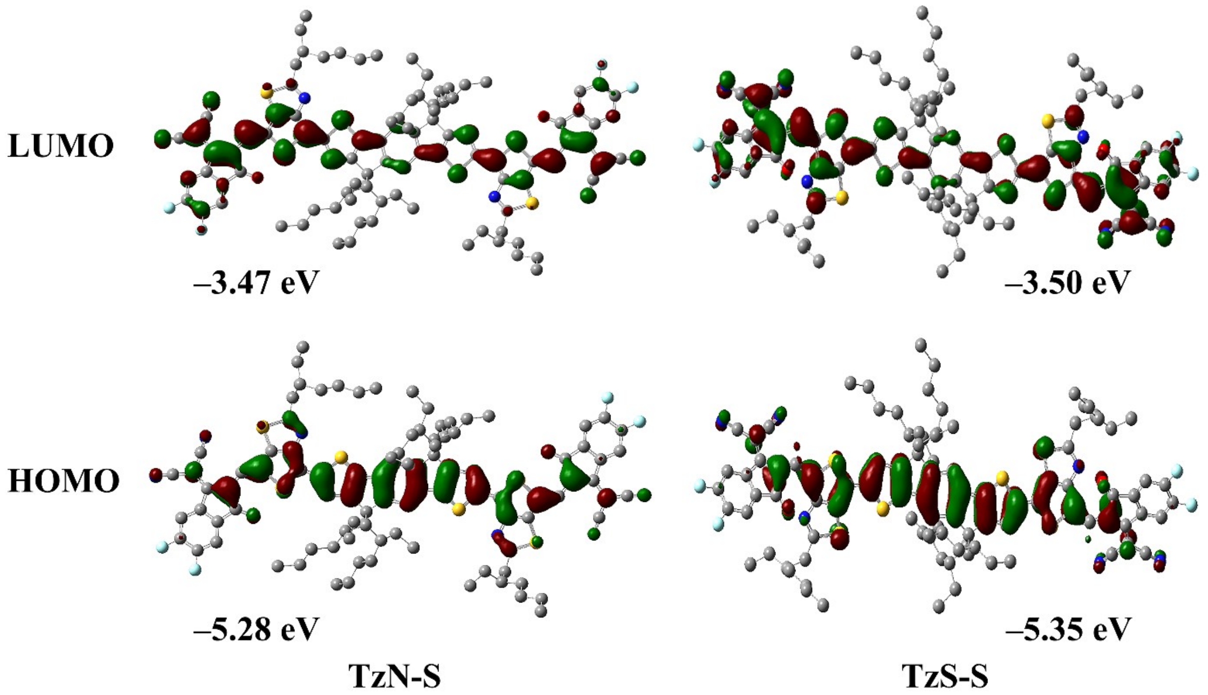

Figure S8. DFT-calculated frontier MOs of newly synthesized regioisomeric NFAs. HOMO seems delocalized over the entire donor backbone while LUMO is localized on terminal acceptor moieties, thereby indicating a smooth flow of charge transfer from donor to acceptor units.

NMR spectroscopic data

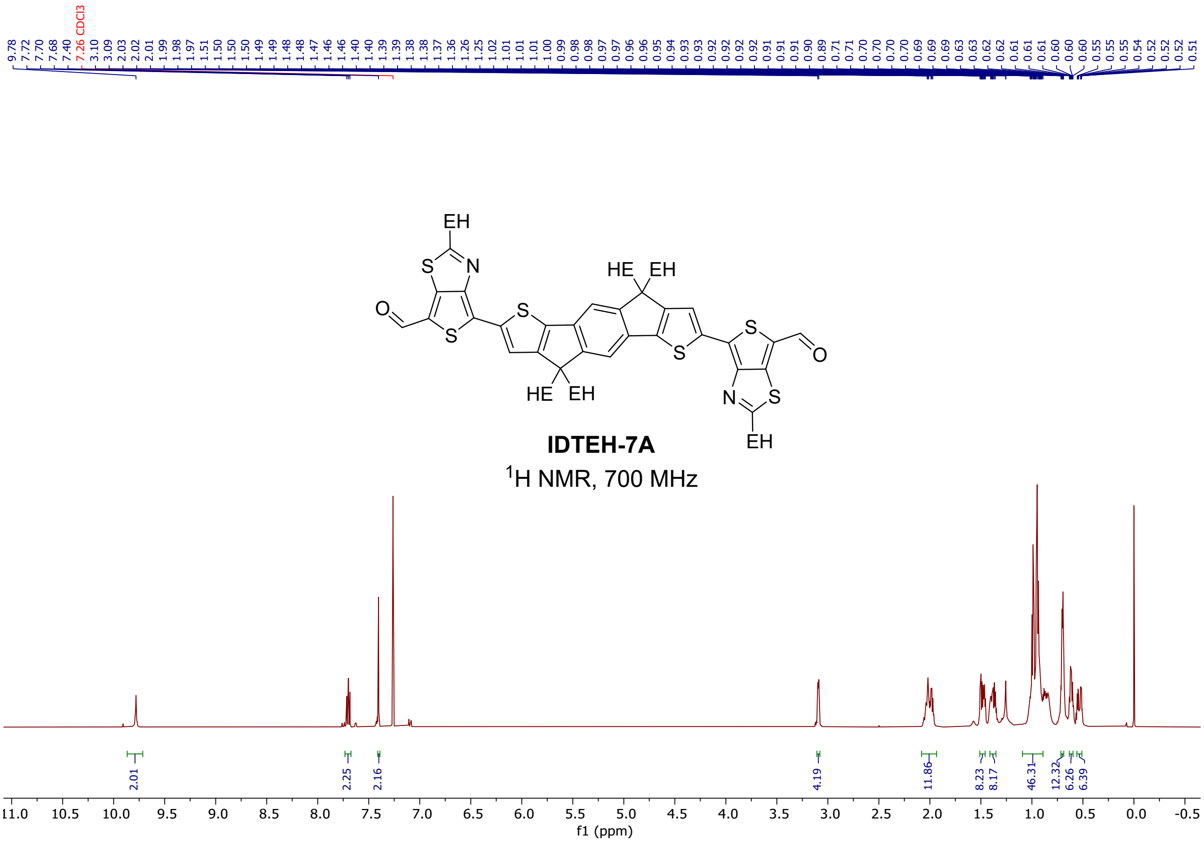

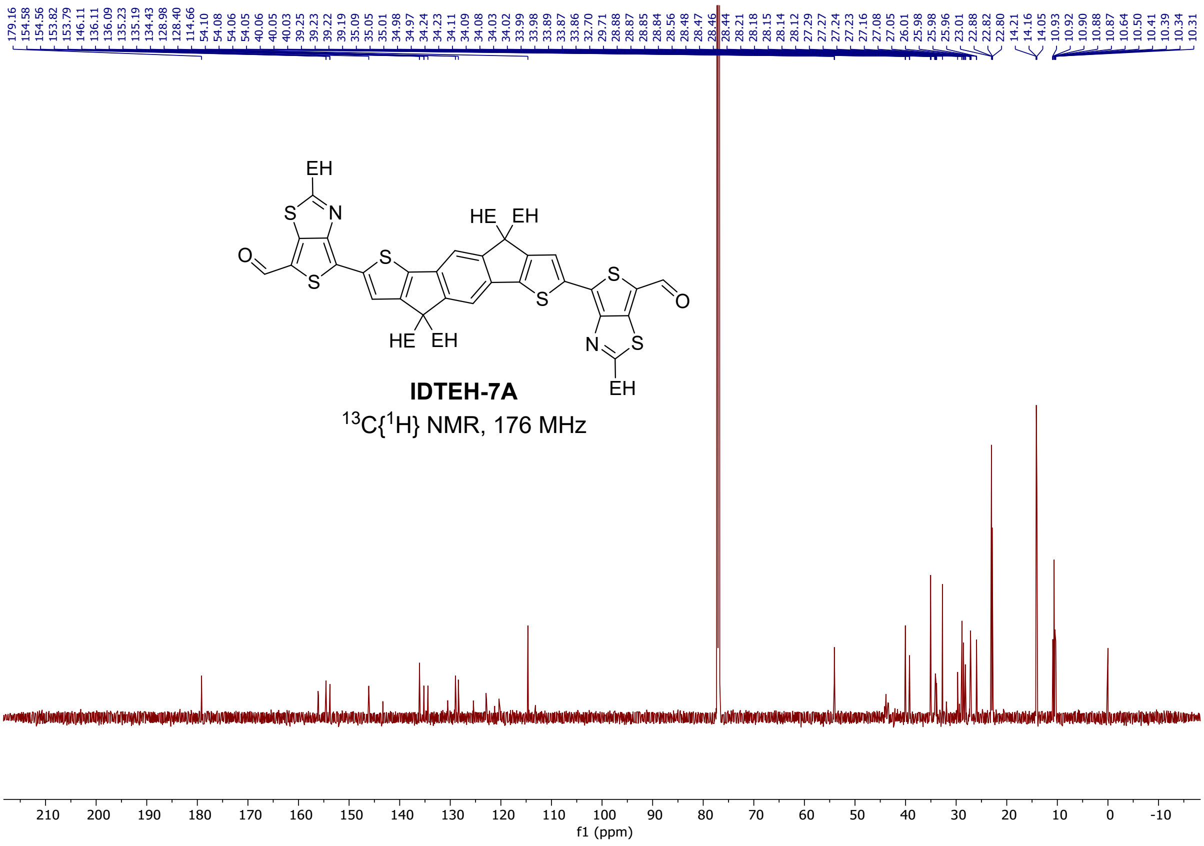

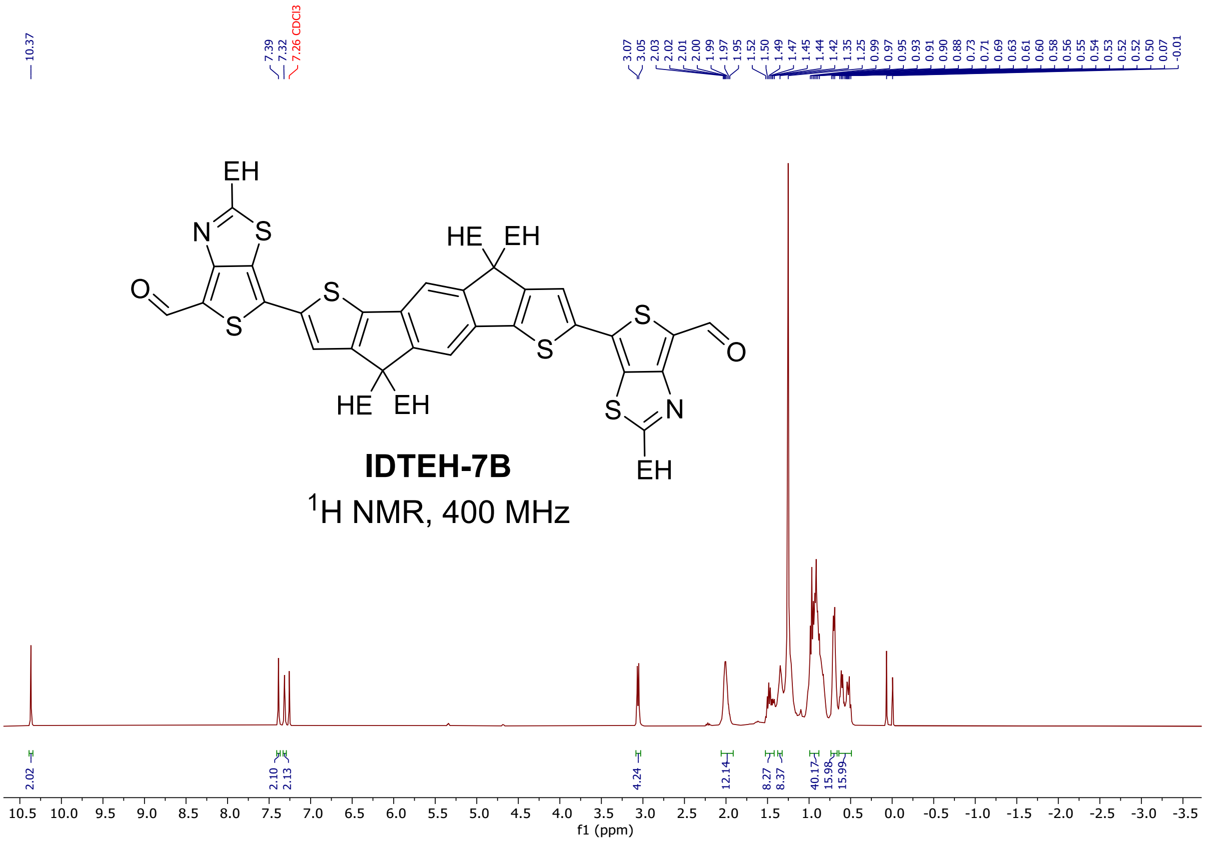
IDTEH-7B
^{1}H NMR, 400 MHz
f1 (ppm)

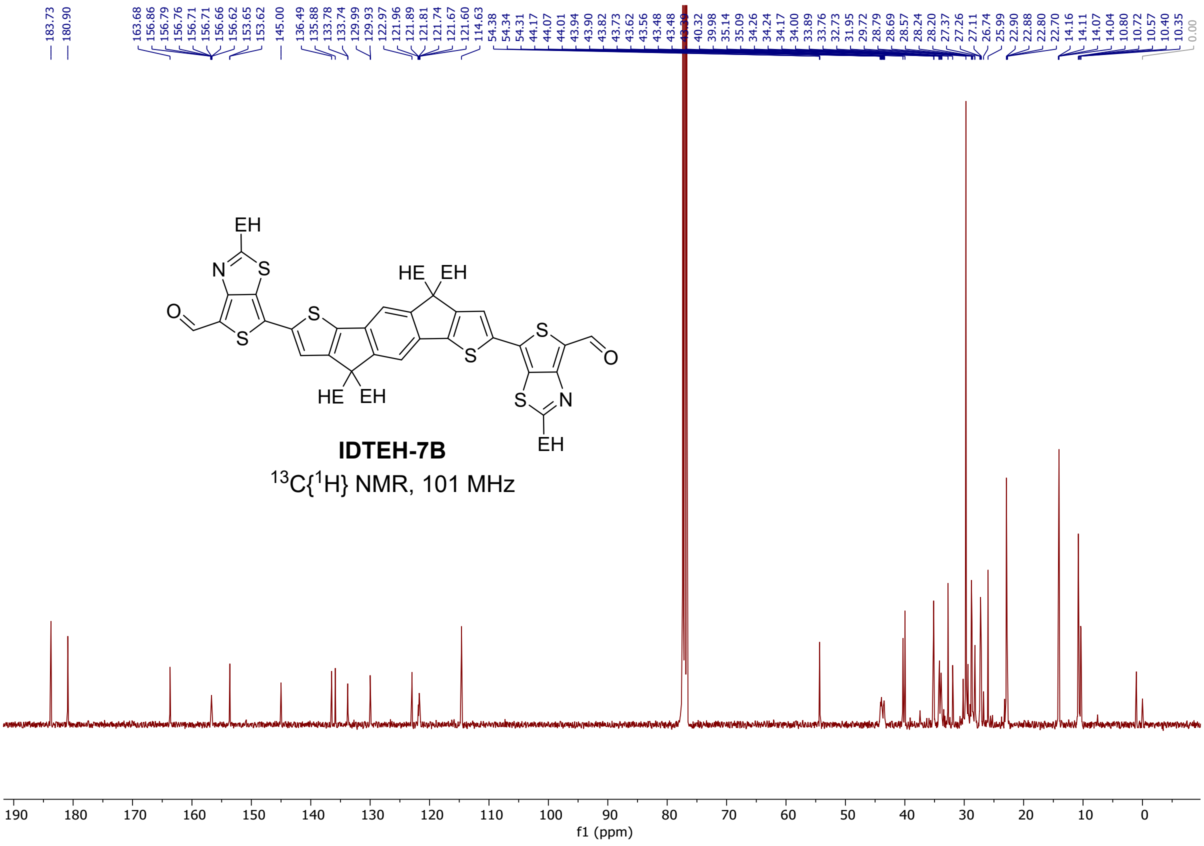
IDTEH-7B
^{13}C{^{1}H} NMR, 101 MHz
f1 (ppm)

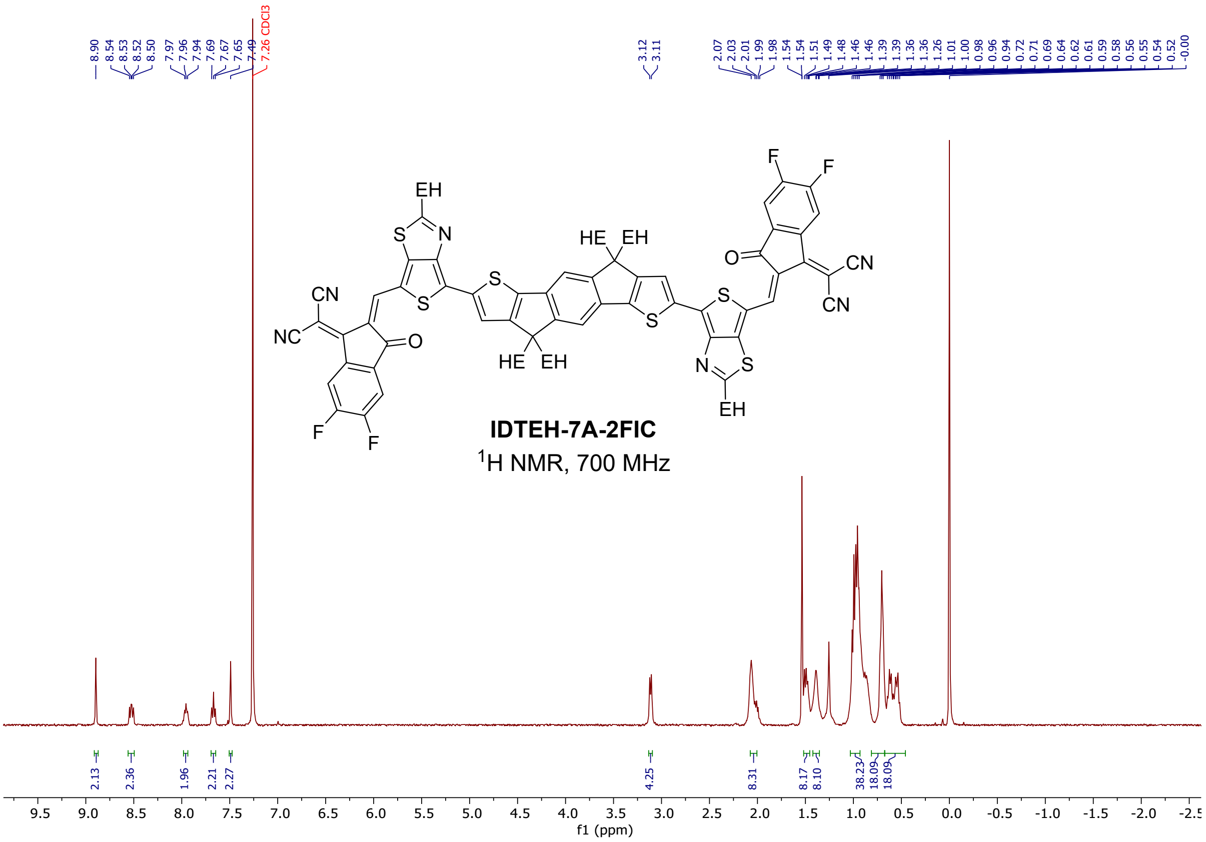
IDTEH-7A-2FIC
^{1}H NMR, 700 MHz
f1 (ppm)

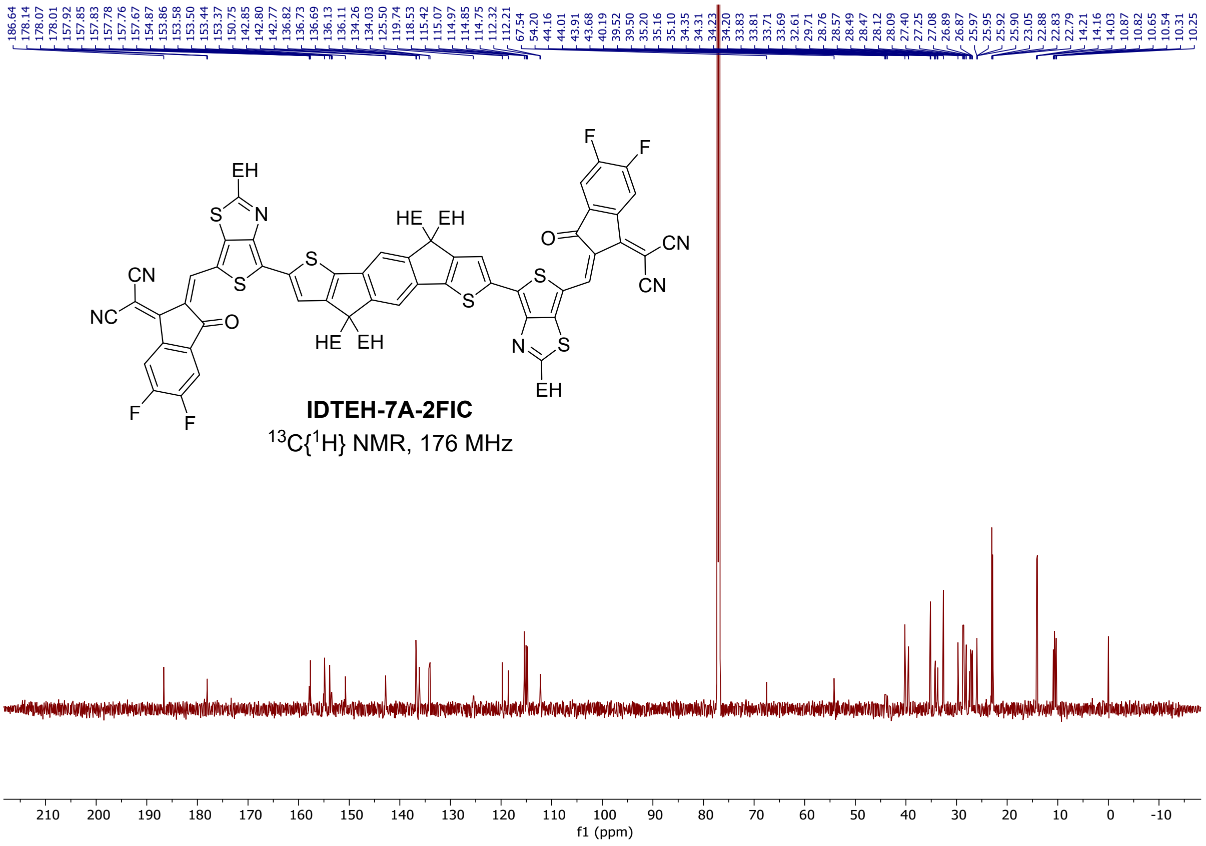
IDTEH-7A-2FIC
^{13}C{^{1}H} NMR, 176 MHz
f1 (ppm)

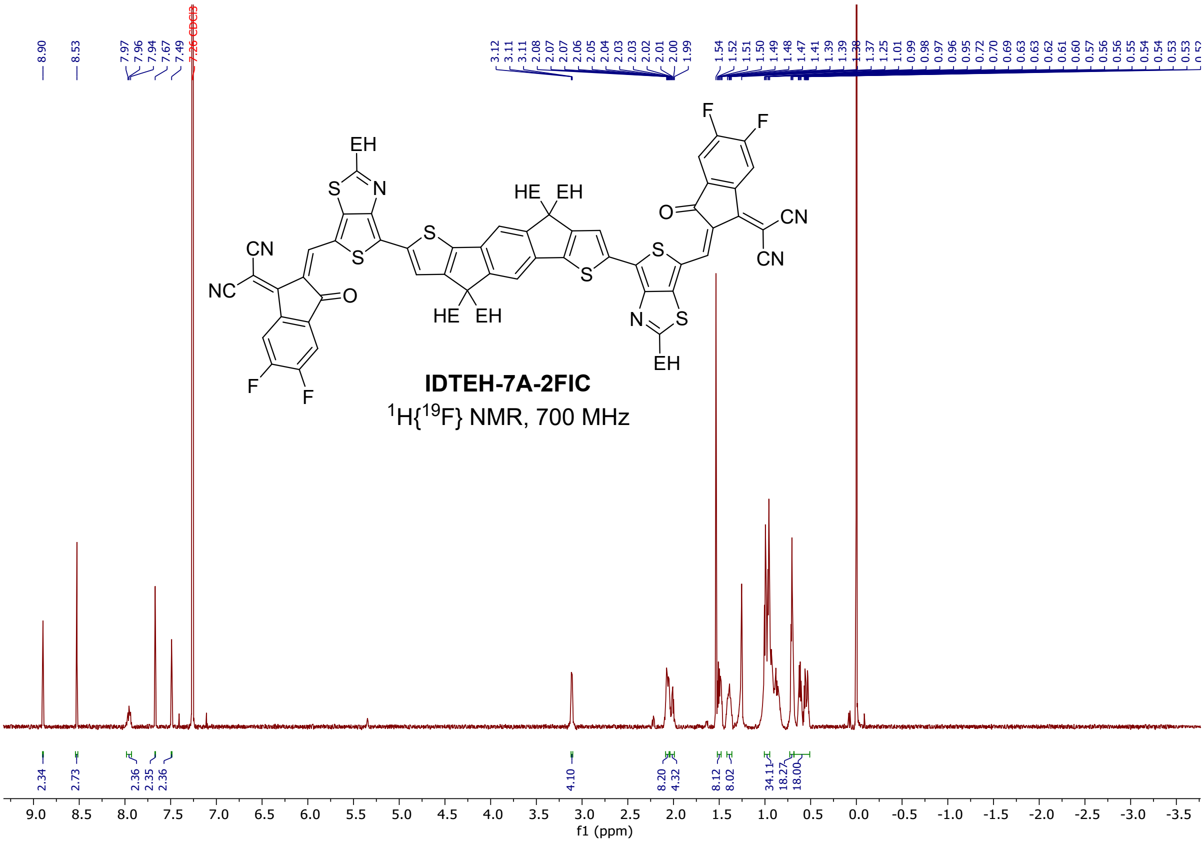
IDTEH-7A-2FIC
$^{1}H\{^{19}F\}$ NMR, 700 MHz
f1 (ppm)

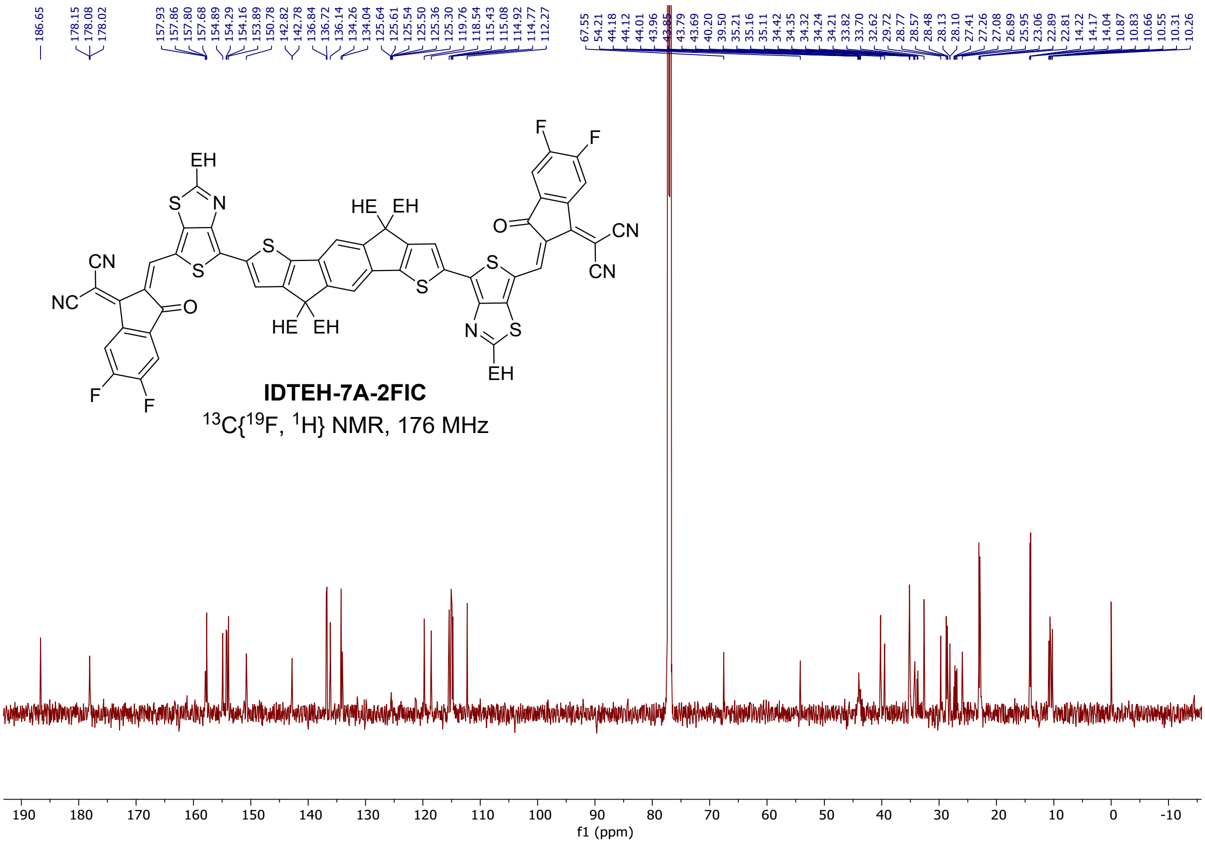
IDTEH-7A-2FIC
$^{13}C\{^{19}F, ^{1}H\}$ NMR, 176 MHz
f1 (ppm)

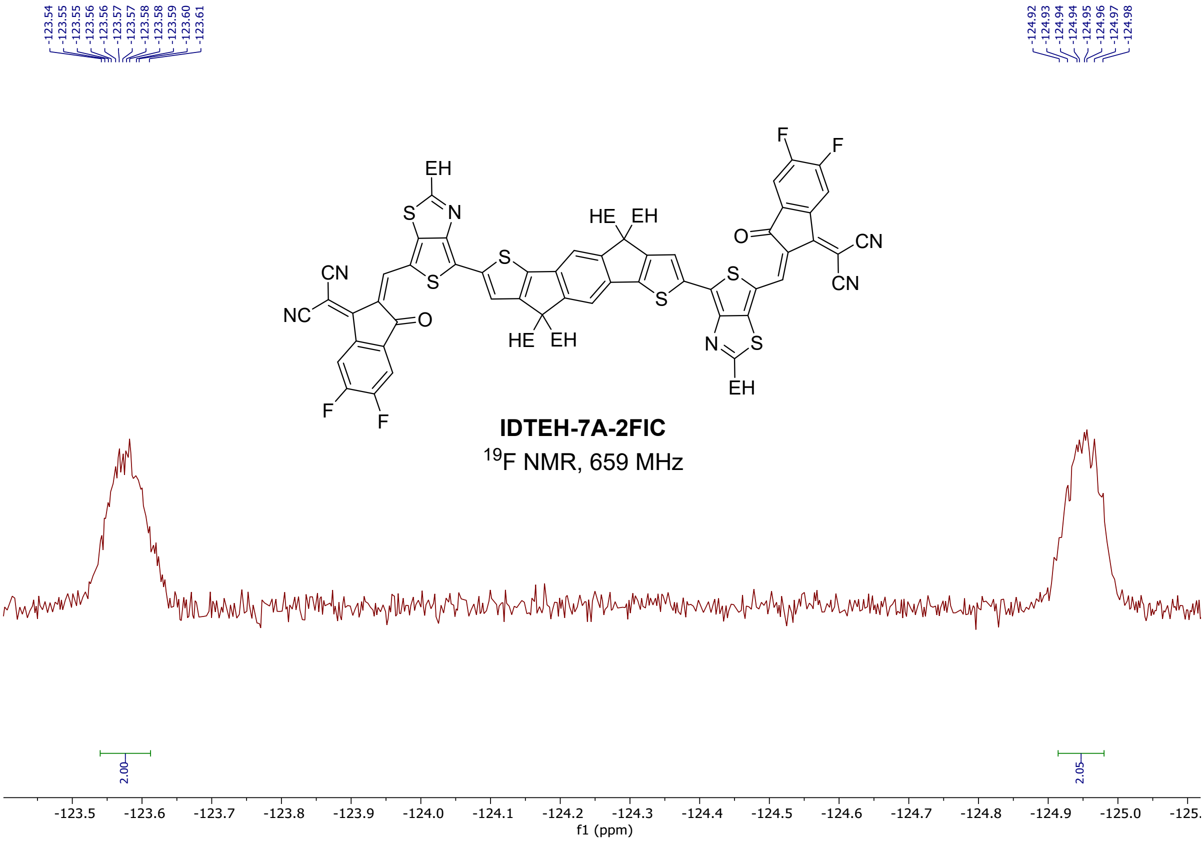
-123.54
-123.55
-123.55
-123.56
-123.56
-123.57
-123.57
-123.58
-123.58
-123.59
-123.60
-123.61
-124.92
-124.93
-124.94
-124.94
-124.95
-124.96
-124.97
-124.98
IDTEH-7A-2FIC
19F NMR, 659 MHz
2.00
2.05
f1 (ppm)

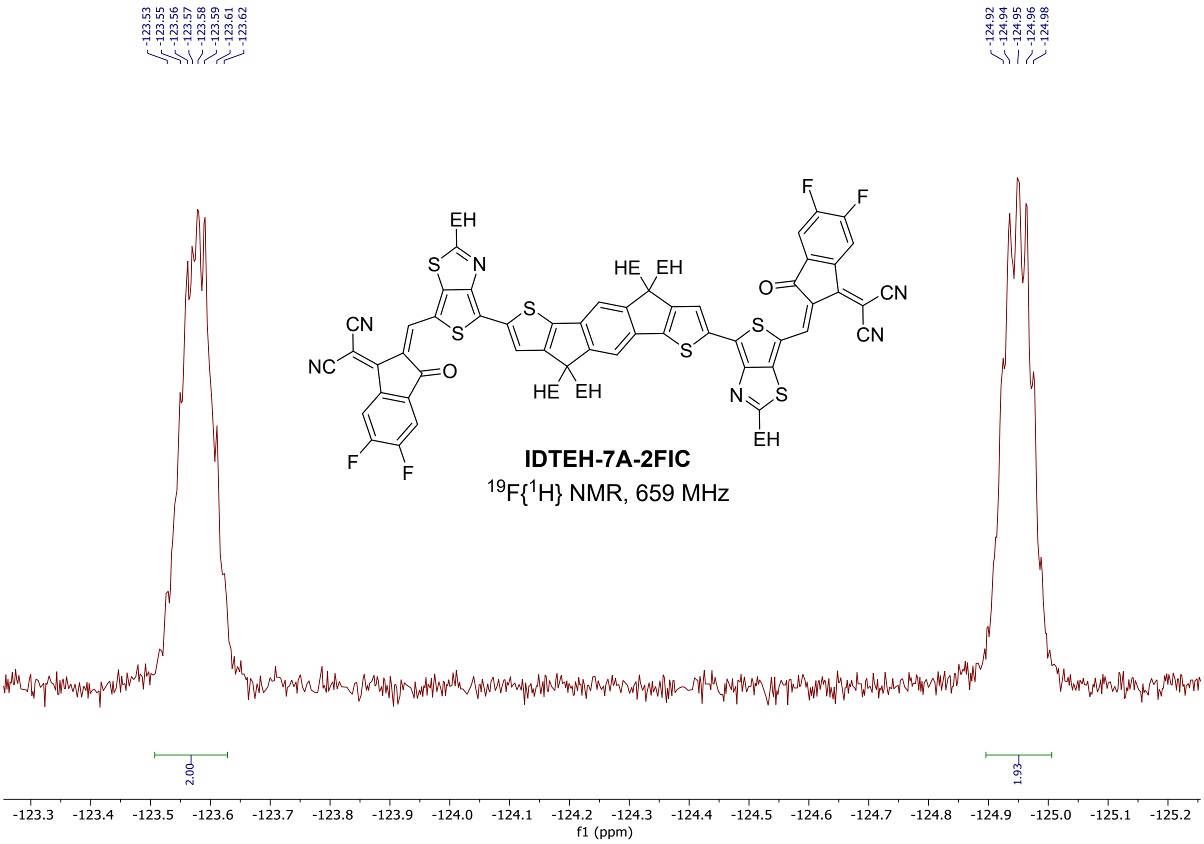
-123.53
-123.55
-123.56
-123.57
-123.58
-123.59
-123.61
-123.62
-124.92
-124.94
-124.95
-124.96
-124.98
IDTEH-7A-2FIC
19F{1H} NMR, 659 MHz
2.00
1.93
f1 (ppm)

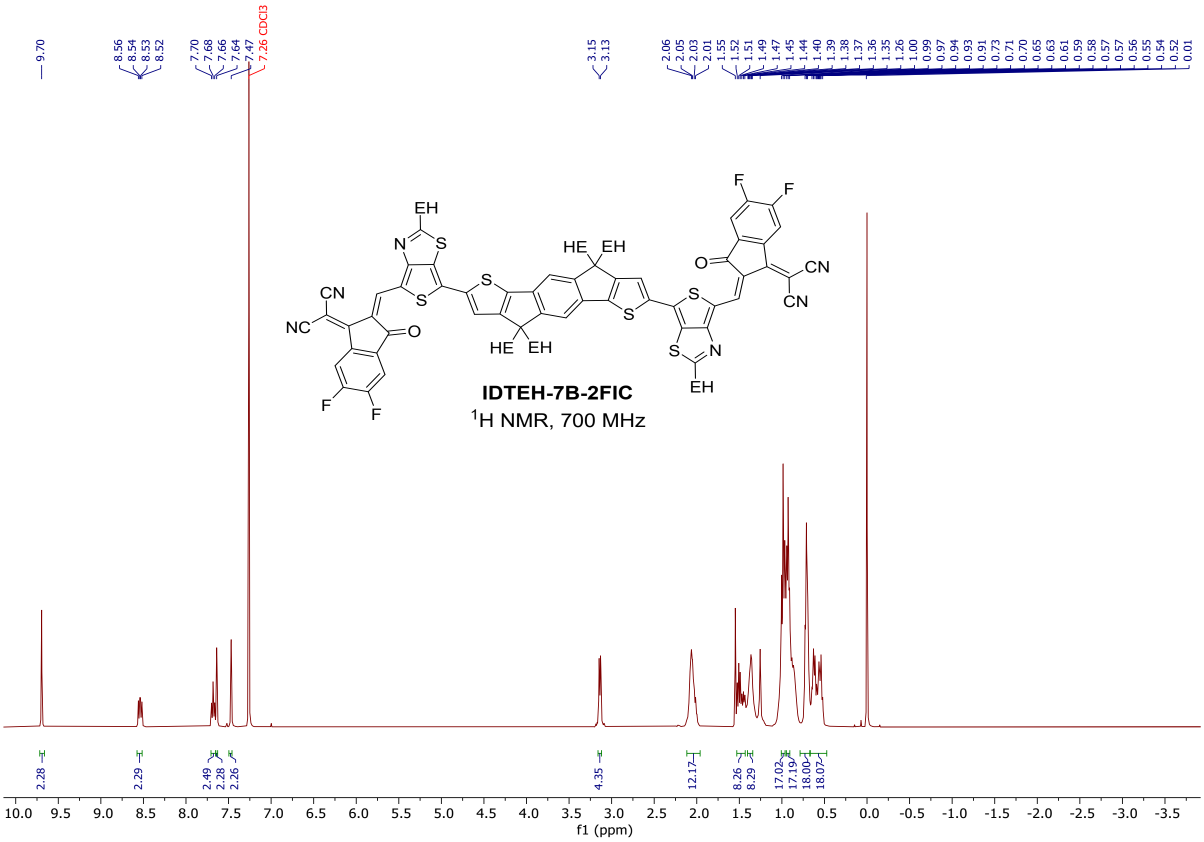
IDTEH-7B-2FIC
^{1}H NMR, 700 MHz
f1 (ppm)

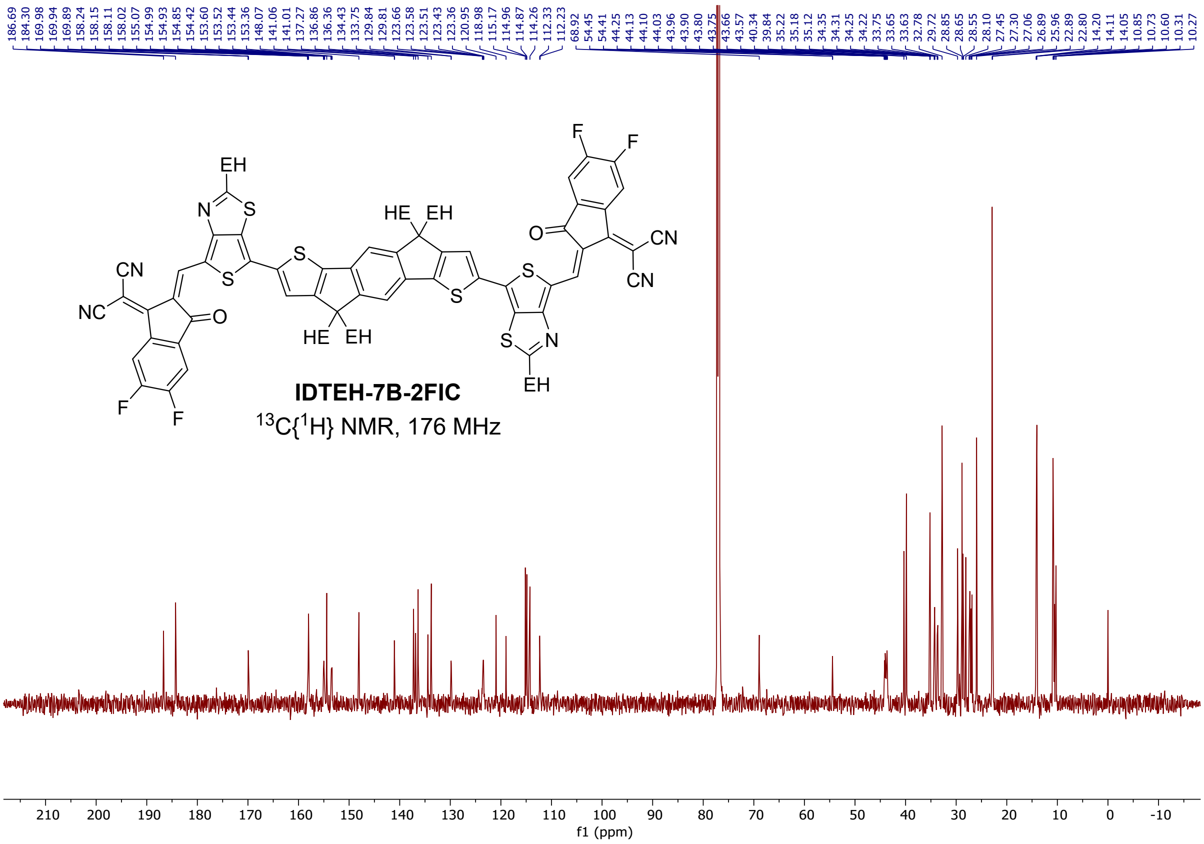
IDTEH-7B-2FIC
^{13}C{^{1}H} NMR, 176 MHz
f1 (ppm)

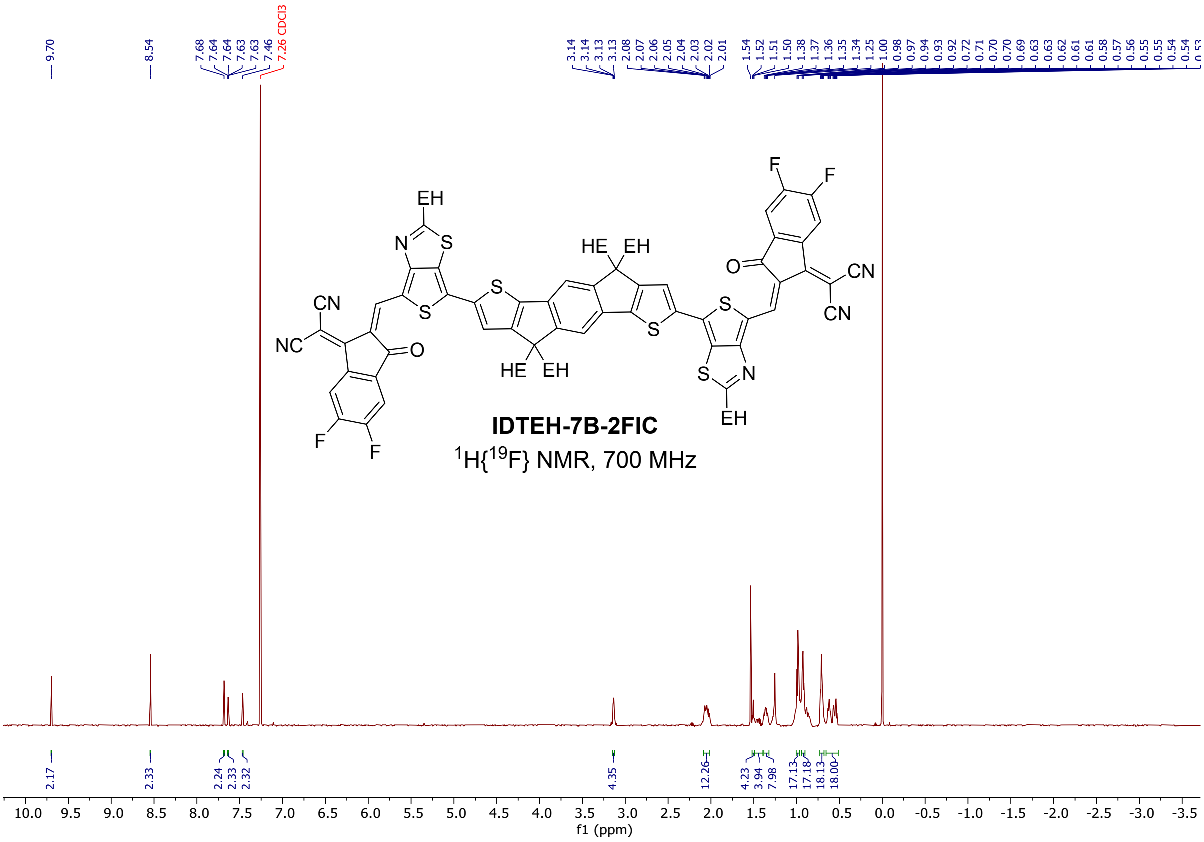

IDTEH-7B-2FIC
^{1}H{^{19}F} NMR, 700 MHz
f1 (ppm)

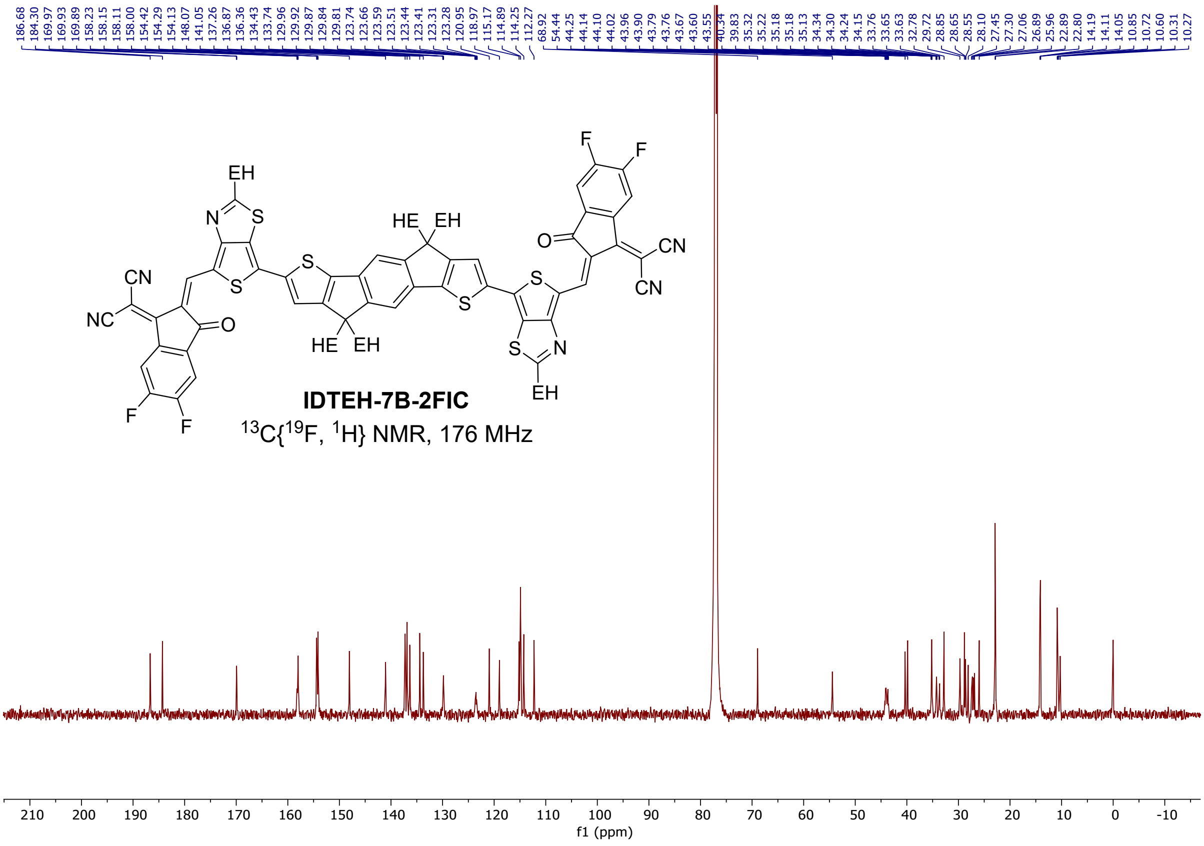

IDTEH-7B-2FIC
^{13}C{^{19}F, ^{1}H} NMR, 176 MHz
f1 (ppm)

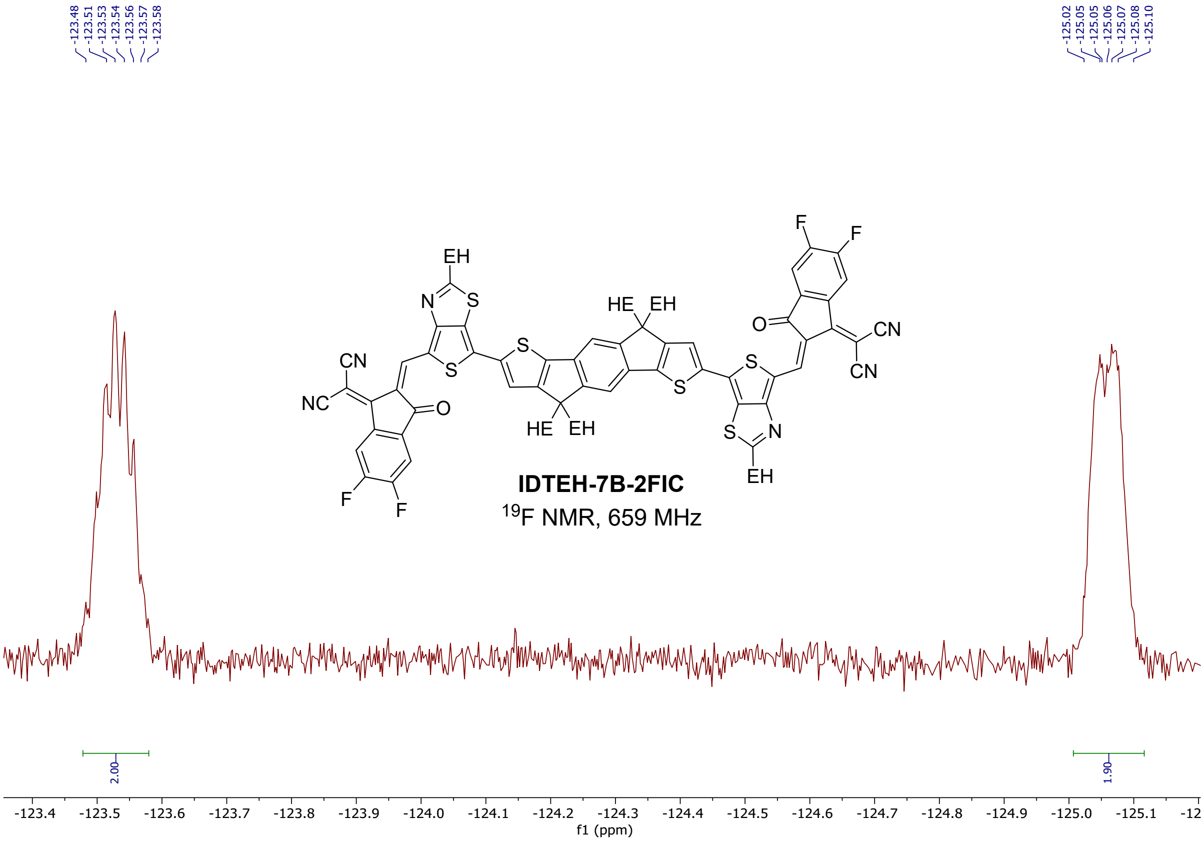
-123.48
-123.51
-123.53
-123.54
-123.56
-123.57
-123.58
-125.02
-125.05
-125.05
-125.06
-125.07
-125.08
-125.10
IDTEH-7B-2FIC
19F NMR, 659 MHz
2.00
1.90
f1 (ppm)

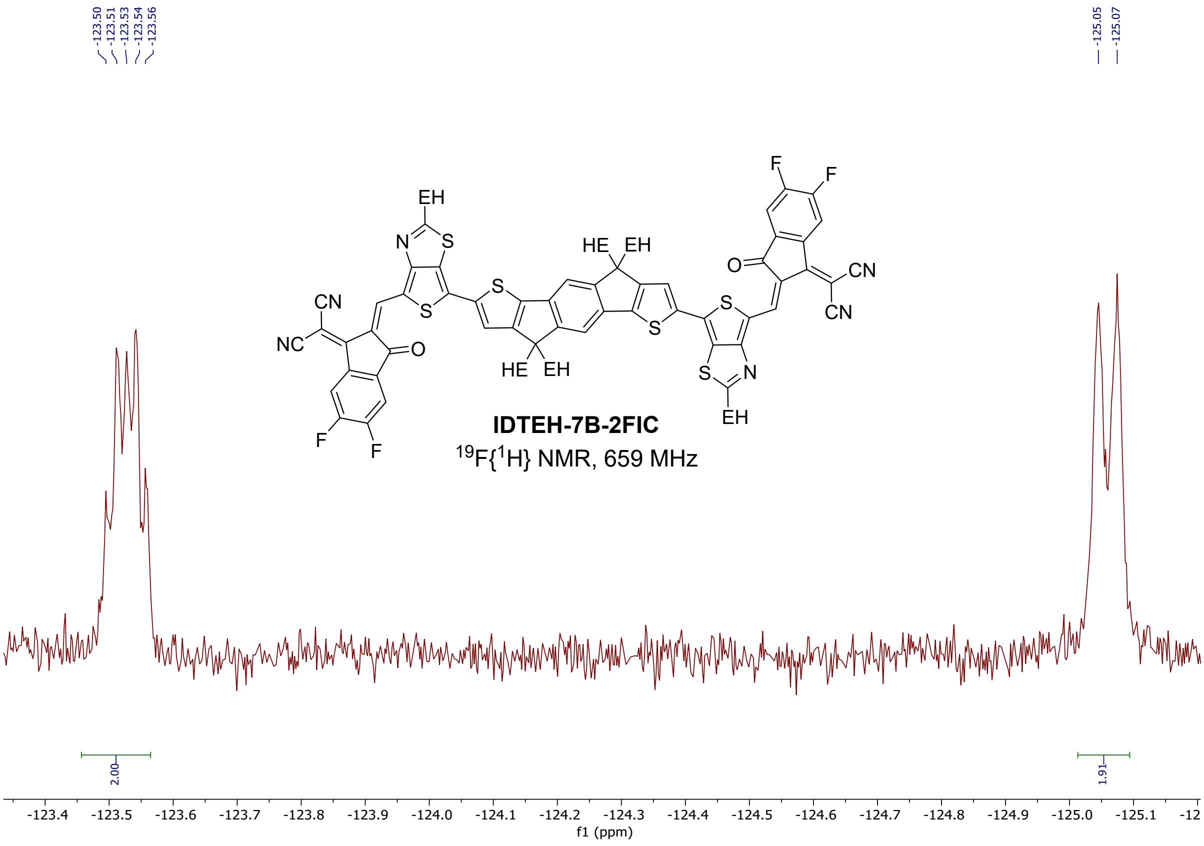
-123.50
-123.51
-123.53
-123.54
-123.56
-125.05
-125.07
IDTEH-7B-2FIC
19F{1H} NMR, 659 MHz
2.00
1.91
f1 (ppm)

References

1. U. Neupane, B. Bahrami, M. Biesecker, M. F. Baroughi and Q. Qiao, *Nano Energy*, 2017, **35**, 128-137.
2. Y. Wang, Z. Zheng, J. Wang, X. Liu, J. Ren, C. An, S. Zhang and J. Hou, *Advanced Materials*, 2023, **35**, 2208305.
3. Z. Zheng, S. Zhang, J. Wang, J. Zhang, D. Zhang, Y. Zhang, Z. Wei, Z. Tang, J. Hou and H. Zhou, *Journal of Materials Chemistry A*, 2019, **7**, 3570-3576.
4. Y. Sun, J. H. Seo, C. J. Takacs, J. Seifter and A. J. Heeger, *Advanced Materials*, 2011, **23**, 1679-1683.
5. X. Hou, D. Chen, Y. Zhang, A. Zhang, K. Zhang, D. Xu and J. Zhang, *Applied Physics A*, 2022, **128**, 708.
6. C. G. Tang, M. C. Y. Ang, K.-K. Choo, V. Keerthi, J.-K. Tan, M. N. Syafiqah, T. Kugler, J. H. Burroughes, R.-Q. Png, L.-L. Chua and P. K. H. Ho, *Nature*, 2016, **539**, 536-540.
7. Z. Zhang, T. Shan, Y. Zhang, L. Zhu, L. Kong, F. Liu and H. Zhong, *Journal of Materials Chemistry C*, 2020, **8**, 4357-4364.
8. Y. Zhang, C. Yu, T. Shan, Y. Chen, Y. Wang, M. Xie, T. Li, Z. Yang and H. Zhong, *Cell Reports Physical Science*, 2022, **3**, 100765.
9. B. Sadowski, M. Loebnitz, D. R. Dombrowski, D. H. Friese and D. T. Gryko, *The Journal of Organic Chemistry*, 2018, **83**, 11645-11653.
10. X. e. Li, Q. Zhang, J. Yu, Y. Xu, R. Zhang, C. Wang, H. Zhang, S. Fabiano, X. Liu, J. Hou, F. Gao and M. Fahlman, *Nature Communications*, 2022, **13**, 2046.
11. S. Braun, W. R. Salaneck and M. Fahlman, *Advanced Materials*, 2009, **21**, 1450-1472.
12. Y. Xie, W. Wang, W. Huang, F. Lin, T. Li, S. Liu, X. Zhan, Y. Liang, C. Gao, H. Wu and Y. Cao, *Energy & Environmental Science*, 2019, **12**, 3556-3566.
13. U. Rau, B. Blank, T. C. M. Müller and T. Kirchartz, *Physical Review Applied*, 2017, **7**, 044016.
